# Twenty Years Later: A Comprehensive Review of the X Chromosome Use in Forensic Genetics

**DOI:** 10.3389/fgene.2020.00926

**Published:** 2020-09-17

**Authors:** Iva Gomes, Nádia Pinto, Sofia Antão-Sousa, Verónica Gomes, Leonor Gusmão, António Amorim

**Affiliations:** ^1^Institute for Research and Innovation in Health Sciences (i3S), University of Porto, Porto, Portugal; ^2^Institute of Molecular Pathology and Immunology, University of Porto (IPATIMUP), Porto, Portugal; ^3^Center of Mathematics, Faculty of Sciences, University of Porto, Porto, Portugal; ^4^Department of Biology, Faculty of Sciences, University of Porto, Porto, Portugal; ^5^DNA Diagnostic Laboratory (LDD), State University of Rio de Janeiro (UERJ), Rio de Janeiro, Brazil

**Keywords:** X chromosome short tandem repeats (X-STRs), X chromosome markers, forensic genetics, population genetics, kinship testing, X chromosome short tandem repeat (X-STR) mutation rates

## Abstract

The unique structure of the X chromosome shaped by evolution has led to the present gender-specific genetic differences, which are not shared by its counterpart, the Y chromosome, and neither by the autosomes. In males, recombination between the X and Y chromosomes is limited to the pseudoautosomal regions, PAR1 and PAR2; therefore, in males, the X chromosome is (almost) entirely transmitted to female offspring. On the other hand, the X chromosome is present in females with two copies that recombine along the whole chromosome during female meiosis and that is transmitted to both female and male descendants. These transmission characteristics, besides the obvious clinical impact (sex chromosome aneuploidies are extremely frequent), make the X chromosome an irreplaceable genetic tool for population genetic-based studies as well as for kinship and forensic investigations. In the early 2000s, the number of publications using X-chromosomal polymorphisms in forensic and population genetic applications increased steadily. However, nearly 20 years later, we observe a conspicuous decrease in the rate of these publications. In light of this observation, the main aim of this article is to provide a comprehensive review of the advances and applications of X-chromosomal markers in population and forensic genetics over the last two decades. The foremost relevant topics are addressed as: (i) developments concerning the number and types of markers available, with special emphasis on short tandem repeat (STR) polymorphisms (STR nomenclatures and practical concerns); (ii) overview of worldwide population (frequency) data; (iii) the use of X-chromosomal markers in (complex) kinship testing and the forensic statistical evaluation of evidence; (iv) segregation and mutation studies; and (v) current weaknesses and future prospects.

## Introduction

**FIGURE 1:**
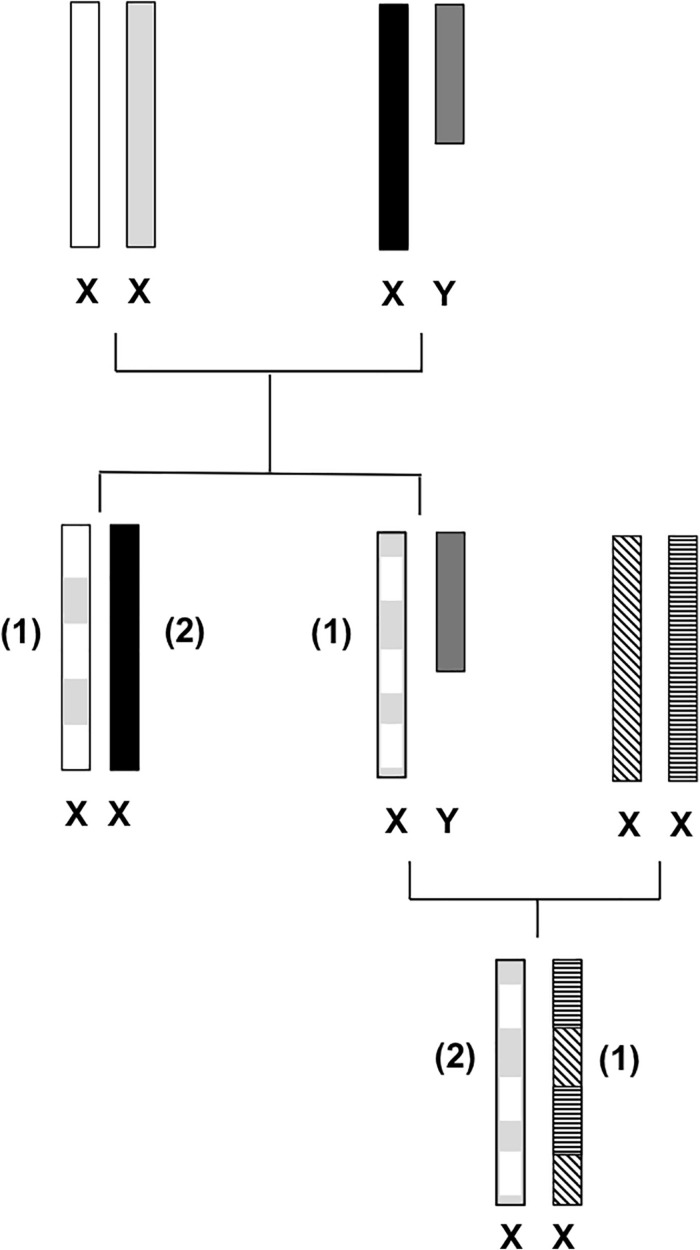
X-chromosomal inheritance. Female and male descendants inherit a recombined maternal X chromosome (1) that resulted from female meiosis. Female offspring inherit one paternal unchanged X chromosome (2) due to lack of recombination [with exception for the pseudoautosomal region (PAR) regions].

The X chromosome has many characteristics that are not shared by its counterpart, the Y chromosome, or by any of the autosomes of the mammalian genome. Its unique structural characteristics have been shaped by evolution, leading to the present known gender-specific genetic differences (Lahn and Page, 1999; Schaffner, 2004). In males, the single copy of the X chromosome does not allow for recombination to occur (except for the pseudoautosomal regions, PARs, which maintain homology by recombining during male meiosis). The non-recombining regions and the PAR 1 and PAR 2 regions of the X and Y chromosomes have taken different evolutionary paths becoming highly differentiated due to different functional roles, and consequently, only a few X-Y sequence similarities remain among them (Lahn and Page, 1999). Mutation events have gathered on the Y chromosome, and in addition to the lack of recombination, these events have contributed to the loss of most of the Y chromosome’s sequence and genes emerging in a distinctive configuration of repeated sequences (Lahn and Page, 1999; Schaffner, 2004) becoming specialized in male sex determination. On the other hand, the X chromosome has preserved its autosomal character, becoming one of the most stable nuclear chromosomes, holding the largest and most conserved gene arrangement across eutherian (“placental”) mammals (Lahn and Page, 1999; Kohn et al., 2004; Schaffner, 2004). It is the only chromosome to have one of its pair inactivated in one sex (females), and it is “corrupted” with repeat elements, making it especially tough to produce a detailed gene sequence (Gunter, 2005). In 2005, Ross et al. (2005) published the first draft that covered approximately 99.3% of the human X chromosome euchromatic sequence. The X chromosome holds a size length of approximately 155 million base pairs (Mb) (Ross et al., 2005), representing nearly 5% of the estimated human genome size (3,200 Mb) (Lander et al., 2001). Regarding some of the X chromosome’s structural properties, it presents a low GC content (39%) when compared to 41% of the genome average (Ross et al., 2005). The low number of functional genes detected confers the chromosome one of the lowest gene densities among the chromosomes annotated to date (Ross et al., 2005). The X chromosome’s sequence data revealed not only a low concentration of genes but also small gene length as only 1.7% of the chromosome sequence is represented by exons of the identified genes, responsible for transcribing 33% of the X chromosome (Ross et al., 2005). The particular genetic characteristics of the X chromosome, shaped by evolution, are responsible for the distinctive gender-specific features (Figure 1): in the male gender, the X chromosome is (almost entirely) transmitted to females as an unchanged block. While in females, the two copies recombine, like autosomes, reorganizing genetic variation in each generation, which contributes to the increase of haplotype diversity (Schaffner, 2004). The new reshuffled chromosome is then transmitted to female and male descendants (Figure 1).

These specific properties – two recombining copies in females and a single non-recombining copy in males (except for the PAR regions) creating haplotypes – provide X chromosome markers a particular place in forensics and in population genetics, as well as in other research areas such as human evolutionary studies and medical genetics (e.g., X-linked recessive disorders such as hemophilia or Duchenne muscular dystrophy) (Szibor, 2007). Regarding forensic and population genetic applications, the X chromosome’s mode of inheritance places this chromosome among the autosomes and the uniparental-inherited genomes [mitochondrial DNA (mtDNA) and Y chromosome] providing desirable and exclusive features that are not provided by any other of the latter.

In the early 2000s, the number of publications using X-chromosomal polymorphisms in these areas of research increased steadily. However, nearly 20 years later, a conspicuous decrease in the rate of these publications is observed. For example, X chromosome short tandem repeat (X-STR) forensic-based publications reached as many as 43 publications in a single year (2009), while in the past year of 2019, only 18 publications were found (complete results and detailed information on the criteria used for database search are presented and discussed under the section “Factors Underlying the Relative Stagnation in X Chromosome Forensic Research”). In light of these observations, the main aim of the present work is to provide an up-to-date and objective review of the advances and applications of X-chromosomal markers in population and forensic genetics over the last two decades since the bloom observed in the early and mid-2000s.

## Current Developments: Numbers and Types of X-Chromosomal Markers Available (Short Tandem Repeats, Single-Nucleotide Polymorphisms, and Insertions/Deletions)

The use of X chromosome polymorphisms in human identification and in population genetics is mainly supported by the potential applications that outcome from its unique properties. Solely, or complementing the information provided by the autosomes or by markers located on the Y chromosome or mtDNA, X chromosome markers may provide essential information in many different lines of research. It must be highlighted that identity testing using X-STRs in particular contexts, namely in scenarios of (complex) kinship testing, may be the only tool to unravel certain cases. Examples of complex kinship testing scenarios where the prominent role of X chromosome polymorphisms is demonstrated are given in the section “The Use of X-Chromosomal Markers in (Complex) Kinship Testing.”

In the present section, we will try to draw the state of the art of the genetic markers that have been described, to date, in the X chromosome-specific region, i.e., leaving out PARs and amelogenin. Special attention is given to X-STRs as a result of their favorite usage in forensic genetics due to high standardization and existence of commercial typing kits. Although some of the first publications reporting X-STRs appeared in the late 90s (Edwards et al., 1991, 1992; Hearne and Todd, 1991; Sleddens et al., 1992), the beginning of the century marked the increase of X-STR publications that focused on the development of new multiplexes on the genetic characterization of many different population groups (databasing) and on kinship and forensic investigations.

An extensive literature review was undertaken, with special focus on forensic-population genetic publications. Results are analyzed and tabulated separately for each type of marker, including relevant references. Supplementary Table 1 lists 85 STR loci in which usage in forensic-population genetic context was reported. In agreement with the study of Szibor et al. (2005), HumARA marker was not considered for ethical reasons. Although the number of X chromosome markers has grown since the 2007 seminal review of Szibor (2007), this growth may be illusory, since many markers were used quite rarely, sometimes only once.

Although a considerable number of X-STRs are available in the literature, a better view of their real, current usage may be given by the analysis of the multiplexes, which have been described for their genotyping. Table 1 shows the most used in-house and commercially developed X-STR multiplexes in which we update the revision of Diegoli (2015) and demonstrate clearly that the effective number of STRs routinely used is modest.

**TABLE 1:** Most used multiplex PCR assays targeting X chromosome short tandem repeat (STR) markers. References do not necessarily refer to the original development papers.

Name	References	Nr. and STR loci
Goldeneye 17X kit	Gao et al. (2019)	16 (DXS6795, DXS9902, DXS8378, HPRTB, GATA165B12, DXS7132, DXS7424, DXS6807, DXS6803, GATA172D05, DXS6800, DXS10134, GATA31E08, DXS10159, DXS6789, and DXS6810)
Investigator^®^ Argus X-12 QS (Qiagen) kit	Elakkary et al. (2014)	12 (DXS7132, DXS7423, DXS8378, DXS10074, DXS10079, DXS10101, DXS10103, DXS10134, DXS10135, DXS10146, DXS10148, and HPRTB)
Microreader^TM^ 19X ID System kit	Lin et al. (2020)	19 (DXS6795, DXS6803, DXS6807, DXS9907, DXS7423, GATA172D05, DXS101, DXS9902, DXS7133, DXS6810, GATA31E08, DXS6800, DXS981, DXS10162, DXS6809, GATA165B12, DXS10079, DXS10135, and HPRTB)
AGCU X19 STR Kit	Li et al. (2017)	19 (DXS8378, DXS7423, DXS10148, DXS10159, DXS10134, DXS7424, DXS10164, DXS10162, DXS7132, DXS10079, DXS6789, DXS101, DXS10103, DXS10101, HPRTB, DXS6809, DXS10075, DXS10074, and DXS10135)
–	Deng et al. (2017)	19 (DXS8378, DXS9898, DXS7133, GATA31E08, GATA172D05, DXS7423, DXS6809,DXS7132, DXS9902, DXS6789, DXS8378, DXS7423, DXS7132, DXS10079, DXS6801, DXS6799, DXS6800, DXS10075, DXS6807, and DXS6803)
–	Prieto-Fernández et al. (2016)	17 (DXS9895, GATA144D04, DXS10077, DXS10078, DXS10161, DXS10160, DXS981, DXS6800, DXS6803, DXS9898, DXS6801, DXS6799, DXS6797, DXS7133, DXS6804, GATA172D05, DXS8377, DXS10146, and DXS10147)
GHEP-ISFG decaplex	Gusmão et al. (2009)	10 (DXS8378, DXS9898, DXS7133, GATA31E08, GATA172D05, DXS7423, DXS6809, DXS7132, DXS9902, and DXS6789)
–	Zhang et al. (2017b)	15 (DXS6807, DXS8378, DXS6795, DXS10164, DXS7132, DXS10074, DXS6803, DXS6801, DXS101, DXS7133, GATA165B12, DXS10103, HPRTB, GATA31E08, and DXS7423)
MiSeq FGx^TM^ Forensic Genomics	Jäger et al. (2017)	7 (HPRTB, DXS7132, DXS7423, DXS8378, DXS10074, DXS10103, and DXS10135)

In any case, due to their high degree of discrimination, the number of standardized STRs is sufficient for most routine investigations, as will be discussed below in the section “The Use of X-Chromosomal Markers in (Complex) Kinship Testing.” Novel interesting STRs for forensic applications continue being described (Nishi et al., 2020). Despite the wide set of available X-STR markers as well as many population-based studies (see section “Overview of Worldwide (Published) X Chromosome Short Tandem Repeat Population Data”) that have emerged over these years, no effective X-STR database exists harboring this type of data. Some of the published population datasets are available in the FamlinkX web page^1^ in a format that can be directly uploaded for kinship calculation using the software. Efforts were made by Szibor et al. (2006) to create an X-STR database^2^ (ChrX-Str.org 2.0, 2020) that could anchor population data (namely, haplotype frequencies), calculation of forensically relevant parameters, information on markers such as multiplex kits, etc. Nevertheless, it seems that no updates have been made to this database, specifically in what regards population data submission, as only four populations are currently available (German, Ghanesen, Japanese, and Chinese Han) (“Haplotypes”; see text footnote 2). In addition, it is however noteworthy that no autosomal STR database such as NIST STRbase (National Institute of Standards, and Technology [Nist], 2020) or STRidER (2020)^3^ contains information on X-STRs either. This approach could be considered: autosomal types of database could potentially serve as harbor for X-STR data undergoing the same quality control (QC) submission criteria. In fact, several forensic-focused journals such as the *Forensic Science International: Genetics* and the *International Journal of Legal Medicine* have published minimum requirements for publication of forensic population data from different genomic markers (e.g., autosomal, Y-chromosomal, mtDNA) (Parson and Roewer, 2010; Gusmão et al., 2017). Submission of such data to these journals requires preliminary QC assessment and inclusion in public online databases. These requirements could certainly be applied to X-chromosomal type of markers, ensuring the same quality type of data submitted. STRs are undoubtedly the preferential markers in human identification applications. Some of the main features that make STRs desirable markers are (i) highly polymorphic, i.e., high discriminating capacity between individuals; (ii) technical easiness due to rapid analysis with PCR-based technology and capillary electrophoresis automated fluorescent detection; and (iii) ability for generating STR multiplexes with small amplicon lengths for degraded DNA. The same cannot be said about insertions/deletions (INDELs), although these share some of the features of STRs (technical ease of analyses by PCR and ability for multiplexing), standardization is much less advanced perhaps due to the need of a much higher number of markers for a high degree of discrimination among individuals. Nevertheless, INDELs represent another potential tool for addressing human genetic identification issues. In Table 2, we list the X chromosome-specific INDEL polymorphisms genotyping systems described in forensic literature.

**TABLE 2:** X chromosome specific insertion/deletion (INDEL) polymorphisms genotyping systems. CE, capillary electrophoresis.

Number of loci	Genotyping system	References
32	Single multiplex (CE)	Pereira et al. (2012)
33	Single multiplex (CE)	Freitas et al. (2010)
16 (from a total of 45 mixed marker system)	Single multiplex (CE)	Tao et al. (2019)
17 (from a total of 60 mixed STR system)	Massive Parallel Sequencing	Zhang et al. (2017b)
21	Single multiplex (CE)	Edelmann et al. (2016)

Unsurprisingly, not as many X chromosome INDEL marker systems have been described as compared to autosomal INDELs (e.g., Pereira et al., 2009; Freitas et al., 2010; Zaumsegel et al., 2013). In fact, no commercial kits being available, few systems have been subject to interlaboratorial comparisons, as in the case of autosomal INDELs, which stood international collaborative exercises (Pereira et al., 2018). One of the possible motifs for the lack of commercial kits is possibly due to the limited applications of X chromosome polymorphisms in forensic genetics when compared to autosomal markers. An interesting alternative typing approach, however, albeit of difficult analysis, is the one described in the studies of Fan et al. (2015, 2016) in which amplicons comprise various INDELs, i.e., biallelic loci that are tightly linked composing a new marker and that are amplified by a single pair of PCR primers.

With respect to X chromosome single nucleotide polymorphisms (X-SNPs), the analysis of the state of the art is even more complex due to the diversity of non-standardized genotyping systems and platforms, which have not been submitted to interlaboratorial comparisons. In Table 3, a summary of the actual forensic use of X chromosome-specific SNPs is shown. The number of table entries gives a false impression of abundance of X-SNPs; in fact, besides the mentioned limitations, the number of SNPs overlap is very low. Although the binary nature of SNPs may favor degraded DNA as well as automation and high-throughput genotyping (e.g., in individual identification using complex kinship analyses in highly degraded scenarios such as natural or human-made disasters), the information content is considerably lower than for STR loci and consequently a larger number of SNPs are needed to match the discrimination power of the commonly used STRs (e.g., Chakraborty et al., 1999; Amorim and Pereira, 2005). Consequently, more loci mean more amplification products, which increases difficulty in data interpretation of DNA profile mixtures. In a multiple-donor sample interpretation, identification of each contributor may be very complex with biallelic systems. The limited number of alleles for each locus (normally two alleles) becomes hard to interpret because overlap will occur and multiple donors become hard to distinguish (Butler et al., 2007; Budowle and van Daal, 2008). Adding the mentioned data interpretation complexity in mixed profiles to the limited applications of X chromosome markers can potentially justify the lack of interest in X-SNPs observed.

**TABLE 3:** X chromosome-specific single-nucleotide polymorphism (SNP) genotyping systems.

Number of SNPs	Genotyping system	References
28 (from a total of 60 mixed marker systems)	Massive parallel sequencing	Zhang et al. (2017b)
39 (from a total of 273 mixed marker panels)	Massive parallel sequencing	Zhang et al. (2017a)
27 (from a total of 1,204 mixed marker panels)	Massive parallel sequencing	Hwa et al. (2018)
62	MALDI-TOF mass spectrometry	Stepanov et al. (2016)
17 (from a total of 220 mixed marker panels)	MALDI-TOF mass spectrometry	Hwa et al. (2019)
5 (from a total of 41 mixed marker panels)	MALDI-TOF mass spectrometry	Petkovski et al. (2005)
10	qPCR (TaqMan probes)	Zarrabeitia et al. (2007)
25	SNaPshot	Tomas et al. (2010)
16	SNaPshot	Oki et al. (2012)
14	qPCR (Taqman probes)	Li et al. (2010)

## Overview of Worldwide (Published) X Chromosome Short Tandem Repeat Population Data

**FIGURE 2:**
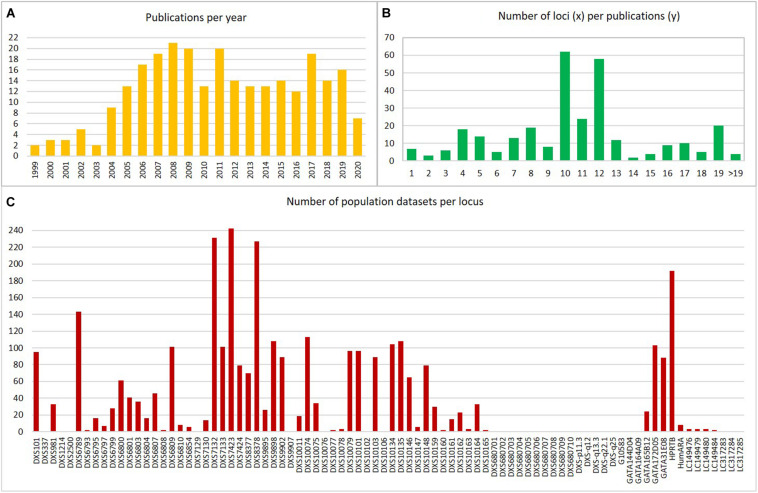
Representation of the compiled human population datasets: **(A)** Number of publications per year; **(B)** Number of loci per population datasets; **(C)** Number of population datasets per locus. Data were obtained from publications referenced in Supplementary Table 2.

For an overview of the worldwide population allele frequency datasets of X-STRs used in forensic genetics, we have consulted the articles available in PubMed database and in the congress proceedings of the International Society for Forensic Genetics^4^ (The International Society for Forensic Genetics [ISFG], 2020). This search resulted in a total of 269 articles. The first genetic studies with focus on genotyping X-STRs for forensic application start emerging in the year 1999. Since then, and until 2008, a remarkable increase of population data publications was observed (Figure 2A). Nevertheless, reported information on human X-STRs in different worldwide populations has been stagnating in the last years.

Information concerning the populations, number of male and female samples, and X-STRs analyzed was compiled using 236 publications out of the 269 consulted (see Supplementary Table 2). The remaining were excluded for different reasons, which include articles that were not in English, with overlapping data (in this case, the most updated dataset was considered), and with unclear information concerning population, markers, or total samples analyzed. Therefore and although some of these studies contain relevant information on X-STR variation (e.g., the study by Edelmann et al., 2006, which has data for DXS9908 and DXS7127 markers), these were not included in Supplementary Table 2. Furthermore, the study by Phillips et al. (2018) reports data on seven X-STRs for a large sample of 944 individuals from the HGDP-CEPH human genome diversity panel. However, since this dataset comprises samples from 51 populations with relatively low sample sizes, the results were compiled for seven continentally defined population groups, namely, African (sub-Saharan), European, Middle East (including North Africans), Central-South Asian, East Asian, Oceanian, and Native American.

In Figure 2B, it is possible to observe that the number of X-STRs analyzed is highly variable among publications with some studies genotyping a high number of X-STRs (e.g., Liu et al., 2013; Fukuta et al., 2019) and others genotyping a reduced number of loci (e.g., Watanabe et al., 2000; Koyama et al., 2002; Carvalho and Pinheiro, 2011). The number of markers included in each study varied from 1 to 27. In 47% of the cases, this number was between 10 and 12 X-STRs, in 31%, it was below 10, and 22% of the datasets included more than 12 makers (Figure 2B). The number of markers available per dataset is somehow related to the use of commercial kits in 37.4% of the population studies (Supplementary Table 2). The first commercial kit that was optimized for forensic applications was the Argus X-UL from Biotype (Dresden, Germany), containing four X-STRs (DXS8378, DXS7132, HPRTB, and DXS7423) located in distant positions along the chromosome to avoid linkage. This kit was soon expanded (Argus X-8) with four additional X-STRs (DXS10135, DXS10074, and DXS10134), creating four pairs of linked X-STRs. The Argus X-12 (Qiagen, Hilden, Germany) is the most recent version of the Argus kit and is the most widely used (an optimized version is now available, the Argus X-12 QS, but that contains the same markers). It comprises 12 X-STRs organized in four linkage groups: LG1, DXS10148/DXS10135/DXS8378; LG2, DXS7132/DXS10079/DXS10074; LG3, DXS10103/HPRTB/DXS10101; and LG4, DXS10146/DXS10134/DXS7423. The Goldeneye DNA ID System 17X (Goldeneye Technology Co., Ltd., Beijing, China) and the AGCU X19 STR kit (Wuxi Sino-German Meilian Biotechnology Co., Jiangsu, China) were also developed for forensic applications, although available data are virtually restricted to Chinese populations. Among in-house multiplexes, the Decaplex system developed by the GHEP-ISFG (Spanish and Portuguese Speaking working group of the ISFG) (Gusmão et al., 2008) has been the most widely used (14.6% of the population datasets were generated using this multiplex).

From the 84 markers that have been described as informative for forensic applications [including HumARA that is no longer used due to ethical issues (Szibor et al., 2005), as already mentioned], less than 50% were studied in more than 10 populations, and 29 were only reported in a single population (Figure 2C). The loci with more allele frequency data accumulated are those included in the commercial kits (namely, Investigator Argus X-12 kit, Qiagen) or in the in-house-developed Decaplex-GHEP-ISFG (Gusmão et al., 2008).

**FIGURE 3:**
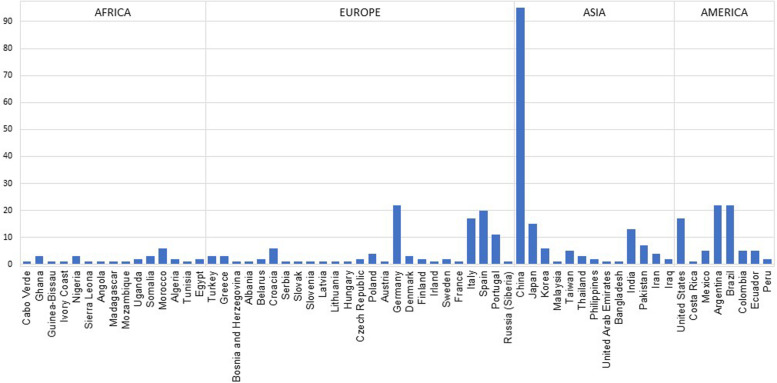
Compilation of number of datasets per country. Data were obtained from publications referenced in Supplementary Table 2.

In Supplementary Table 2, the geographical distribution of the published human population data for X-STRs since 1999 is described. Notwithstanding the exhaustive nature of this review, it is possible that some studies are missing from this table. However, we believe that most forensic population studies on X-STRs have been identified, allowing a realistic picture of the state of the art. For a broader overview of the populations sampled, we have represented the number of datasets that have been published until now by country (Figure 3). The datasets were counted considering the number of subpopulations or ethnic groups in each publication. Populations defined at continental level (namely, the HGDP-CEPH and Africa datasets) or belonging to ethnic affiliated populations from different countries (namely, the Jews) have been excluded. In Figure 3, it is possible to observe that apart from a lack of X-STR data information for many countries, there is high heterogeneity among and inside continents. Data are scarcer in some geographical areas, namely, for sub-Saharan African and American populations (except for Argentina, Brazil, and United States). On the other hand, a large quantity of X-STR data was obtained for other populations, such as the ones from China. China is by far the best represented country not only because of the higher number of publications but also due to the inclusion of various ethnic groups in a single study. Although for some countries a large number of datasets are available for the same X-STR loci, many of those studies characterize different regions or subpopulations, which is relevant to investigate population stratification inside the country, especially when a high diversity of ethnicities coexists.

Overall, the compiled information clearly shows an imbalance between the total number of publications and the asymmetric representation of the worldwide populations. In fact, for several populations from different geographic regions, data on X-STR remain largely scarce, being the available information representative of only a small fraction of the worldwide human populations. Moreover, apart from a large variation concerning the X-STRs included in each study, many only comprise a small number of loci. Due to proximity on the chromosome, it is expected that some of the studied markers will be in linkage disequilibrium (LD) in many populations. However, data on haplotype frequencies are almost restricted to recent papers and not available for most publications consulted, invalidating the use of some of the available data in forensic applications.

Therefore, further studies on haplotype frequency distributions, as well as on mutation rates and LD, are mandatory to attain the final goal of establishing highly comprehensive and representative human reference X-STR databases.

## Short Tandem Repeat Nomenclatures and Practical Concerns

Accuracy and common nomenclature are of fundamental importance to secure error-free communication, data exchange, and data comparison among laboratories. STR nomenclature, independently of marker genome location, has been long addressed by several studies (e.g., Lazaruk et al., 2001; Gusmão et al., 2002; Gomes et al., 2008, 2009, 2016; Gettings et al., 2015) as well as by the ISFG and other DNA groups (e.g., Bär et al., 1997; Olaisen et al., 1998; Gill et al., 2001; Gusmão et al., 2006).

The observed increase of X-STR studies over the years justifies the need to evaluate X-STR nomenclature being used at least for the most common polymorphisms. Several studies have gathered considerable sequencing data for some of the commonly used X-STRs (Gomes et al., 2008, 2009, 2016, 2017; Szibor et al., 2009). In these latter studies, relevant findings were reported for several markers, which demonstrate that accurate allele nomenclature designation taking into consideration the ISFG recommendations (Gusmão et al., 2006) would have had a major impact on allele assignment. One of the major gaps seen in several studies is the lack of sequencing data for, at least, the three major population groups (Asian, African, and Caucasian) when new markers are proposed as usually only one group is analyzed. This approach reduces possible interpopulational variation and avoids genotyping problems when different groups are genotyped. This was the case for the first version of the most used X-STR commercial kit, the Investigator Argus X-12 (Qiagen). The markers in this kit were characterized mostly in individuals of European ancestry and therefore some of the genetic variations detected in other population groups were missed out (Tillmar et al., 2017). Once other population groups of other ancestries were studied, several markers presented high frequencies of silent alleles that had gone previously undetected (e.g., Tomas et al., 2012; Gomes et al., 2016, 2017; Tillmar et al., 2017). For example, the silent alleles for some of the loci were mostly caused by a mismatch at one of the primer binding sites (Gomes et al., 2016, 2017). After several reports on this matter, a new version was developed, the Investigator Argus X-12 QS (Qiagen), containing the same markers but with new primer designs for some of the X-STRs to resolve the high frequency of allele dropouts. Another example of inaccurate nomenclature assignment was the case of HPRTB. In the study of Pereira et al. (2007), peculiar results during population comparison analyses of a Northern Portuguese population sample with other European groups were found. These findings led to a deeper investigation, leading to the discovery of issues behind the HPRTB nomenclature (Szibor et al., 2009). In this latter report, authors described that two different nomenclatures were being used among the forensic genetic community, leading to a shift in allele frequencies and consequently errors in data resulting from population comparisons-based analyses (e.g., Pereira et al., 2007).

Finally, as proposed by the ISFG recommendations on the use of X-chromosome markers (Tillmar et al., 2017), the previous recommendations on allele nomenclature already recognized for autosomal and Y-chromosomal-specific markers (Bär et al., 1997; Gill et al., 2001; Gusmão et al., 2006) can also be applied to X-STRs without the need for particular changes. It seems that very few studies take these recommendations into thoughtful consideration and no real significant advances have been made in this field. Accuracy in sequence variation and repeat structure and nomenclature of X-STRs are empirical and pending issues in forensic and population genetics research that are still often neglected.

## The Use of X-Chromosomal Markers in (Complex) Kinship Testing

The standard procedure to quantify the genetic evidence in kinship analyses relies upon independent autosomal markers and is grounded in Bayes’ theorem. Typically, equal priors are considered, and a likelihood ratio (LR) comparing the probability of the observations assuming a pair of alternative, mutually exclusive, kinship hypotheses is computed (Gjertson et al., 2007). Indeed, autosomal information is the one generally considered, despite currently available X-chromosomal markers being able to provide great statistical power in some cases (Szibor et al., 2003; Krawczak, 2007; Szibor, 2007; Pinto et al., 2011a, 2013a; Gomes et al., 2012). From the set of the latter cases obviously excluded are those where there is a link “father–son” in both main and alternative hypotheses, as, for instance, in a “paternal grandfather–granddaughter” vs “unrelated” case analyzing a pair of individuals, as the first, when considering X-chromosomal transmission, equated to the second (Pinto et al., 2011a, 2012). In any case, the preference given to autosomal markers is easily justified and understood not only for allowing the same approach for each kinship problem, regardless of the sex of the involved individuals, but also because of independent transmission of the markers and, at least in most of the populations, absence of LD. Conversely, the analysis of X chromosome markers offers little room to consider only independently transmitted loci, and thus recombination rates and haplotype frequencies are in general required for statistical evaluation of the evidence.

Non-random association of alleles of different loci at a population-level LD (also known as gametic association) can result from population events like drift, selection, non-random mating, or admixture (Hedrick, 1987; Medina-Acosta, 2011). A close physical location of the markers, as well as population stratification, will influence the re-establishing of equilibrium. Consequently, LD results neither can be extrapolated from one population to another, nor are stable, even in a closed population, as recombination progressively breaks it. Moreover, haplotype frequencies cannot be inferred from allelic ones, and direct counting needs to be carried out.

Closely located markers are said to be in linkage if they are more prone to be inherited together, as a unit, than independently. Linkage between markers depends on chromosomal recombination rate (or frequency). Two markers are unlinked if recombination between them is expected to occur in each meiosis so that half of the gametic products would be recombinant and thus recombination fraction takes the value of 0.50. Obviously, linked markers are more prone to be in LD. Segregation analyses in one or multi generation family studies were performed, aiming to estimate recombination rates between X-STRs of interest through proper bioinformatic pipelines that take into account the possibility of mutation (Nothnagel et al., 2012; Diegoli et al., 2016; Bini et al., 2019), but population-based studies, as HapMap project (The International HapMap Consortium, 2007), can also be considered (Phillips et al., 2012). Mapping functions as Haldane’s (Haldane, 1919) or Kosambi’s (Kosambi, 1944) are used to convert genetic distances between markers in recombination rates. It is however noteworthy that in some kinship problems, as the one involving a pair of females and the hypotheses maternity and unrelated, the linkage is not needed to be taken into account as it cancels in the LR numerator and denominator (Tillmar et al., 2017). A general framework to understand in which case linkage has to be considered is still lacking, despite being known that disregarding it may lead to a significant over- or under-quantification of the genetic evidence (Tillmar et al., 2011; Kling et al., 2015b).

Contrarily to what occurs for autosomes, where a plethora of markers from 22 chromosomes can be chosen, linkage and LD are unavoidable issues in the case of X-chromosomal analysis. Due to the length of the X chromosome, a maximum of four unlinked X-STRs are estimated to be liable of being simultaneously analyzed. On the other hand, higher LD values are expected for X-chromosomal markers than for autosomes since recombination only occurs in female meioses, which have also smaller mutation rates than males (Shimmin et al., 1993; Schaffner, 2004). Finally, it should be noted that estimates of haplotype frequencies are not as accurate as the allelic ones since much larger databases are required: just considering a simple illustrative example, a set of three loci with 10 alleles each can potentially entail the estimation of 1,000 haplotype frequencies.

Few software packages are available for kinship evaluations considering X-chromosomal transmission, FamLinkX being the most relevant, taking into account the possibility of mutation, linkage, and LD (Tillmar et al., 2011; Kling et al., 2015a). Also, software to weigh the *a priori* power of a marker to exclude a claimed relationship was already developed (Egeland et al., 2014), and the ISFG recently provided general guidelines for using X-chromosomal markers in kinship testing (Tillmar et al., 2017).

### Kinship Testing and the Identity-by-Descent Framework

Considering a number of generations beyond which individuals are assumed to be unrelated, kinship measurements are based on the concept of identity-by-descent. Two alleles are called identical-by-descent (IBD) if they are copies of a given ancestral allele. Barring mutation, two alleles which are identical by descent must be therefore identical-by-state (IBS). For autosomal transmission, nine IBD partitions can be established considering the four alleles of a pair of individuals and their relationship (Jacquard, 1974; Weir et al., 2006; Pinto et al., 2010). This number reduces to three if non-inbred individuals are considered, likewise occurring for X-chromosomal transmission between a pair of females (Pinto et al., 2011a, 2012). Regarding X-chromosomal transmission, there are four IBD partitions involving a female–male pair (two if assuming a non-inbred female) and two for a pair of males (Pinto et al., 2011a). Independently of the mode of genetic transmission considered, the probabilities of the genotypic observations, assuming a specific hypothesis of kinship, depend on the IBD probabilities of the pedigree and on the frequency of the alleles (Weir et al., 2006; Pinto et al., 2011a). Pedigrees with the same IBD coefficients are said to belong to the same kinship class, as they are, theoretically, undistinguishable through the use of unlinked markers (Pinto et al., 2010, 2012). In Table 4, IBD probabilities are presented for a pair of non-inbred individuals considering autosomal and X-chromosomal modes of genetic transmission and a set of commonly analyzed relationships. Algebraic formulae for the probabilities of the observations, given the identity by descent partitions, can be found in Weir et al. (2006) and Pinto et al. (2010, 2011a, 2012), respectively, for autosomes and X-chromosomal markers. Finally, it should be noted that, assuming X-chromosomal mode of transmission, relationships are not symmetrical as probabilities of IBD sharing may differ. For example, while a pair of paternal aunt–nephew does not share X-IBD alleles (being thus equated to unrelated from the X-chromosomal point of view), a pair of paternal uncle–niece shares one pair of IBD alleles with 50% of chance.

**TABLE 4:** Probability of two individuals sharing two, one, or no pairs of identical-by-descent (IBD) alleles, assuming a specific kinship for both autosomal (Aut) and X-chromosomal (X chr) modes of genetic transmission.

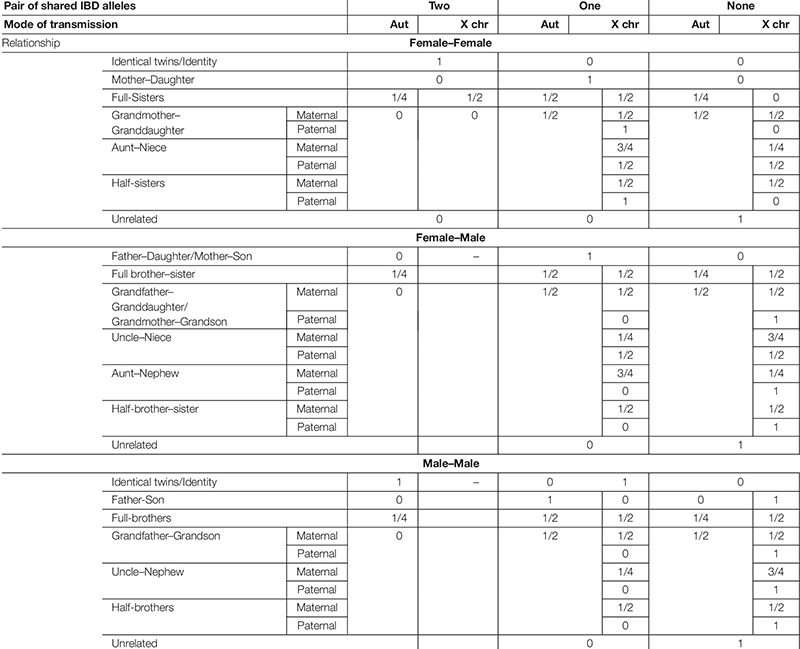

Regardless of the mode of genetic transmission considered, striking statistical results could be obtained when the sharing of IBD alleles is mandatory, unless mutation occurs, for one of the two kinship hypotheses considered. For example, in a standard paternity problem (“unrelated” as alternative hypothesis), the probability of sharing a pair of IBD autosomal alleles (and thus IBS, barring mutation) is one, under the main hypothesis, and null under the alternative. In cases with daughters, this is also true for X-chromosomal markers, providing a higher *a priori* paternity exclusion power than autosomal ones (Krawczak, 2007; Pinto et al., 2013a).

In some cases, as in disaster victim identification problems, specific kinship hypotheses cannot be established, and a broader measure of kinship can be established to weigh the degree of relatedness before specifying more detailed hypotheses. In these cases, the coancestry coefficient, i.e., the probability of selecting two IBD alleles when each one is randomly chosen from each individual, can be computed. In this case, the analysis of the X chromosome can be of major importance as, in all the cases where transmission is not interrupted by a “father–son” link, the expected IBD sharing is at least the same as for autosomes – see Table 5, since no randomness is possible in the X-allele of a male. Coancestry coefficients can be estimated through the genotypes of the individuals (Pinto et al., 2011b, 2013b) and the combination of both types of genetic information can provide valuable insights on the genetic kinship linking the individuals.

**TABLE 5:** Probability of choosing a pair of identical-by-descent (IBD) alleles when one allele is randomly chosen from each individual. Numbers in superscript in header refer to the sex of the individuals represented in genealogies.

Kinship		Coancestry	Female^1^–Female^2^	Male^1^–Male^2^	Female^1^–Male^2^	Male^1^–Female^2^
General		Aut-chr	1/2k_2_ + 1/4k_1_			
		
		X-chr	1/2x_2_ + 1/4x_1_	x_1_	1/2x_1_	1/2x_1_

Parenthood		Aut-chr	1/4			
		
	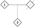	X-chr	1/4	0	1/2	1/2

Full-sibship		Aut-chr	1/4			
		
	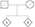	X-chr	3/8	1/2	1/4	1/4

Paternal half-sibship		Aut-chr	1/8			
		
	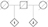	X-chr	1/4	0	0	0

Maternal half-sibship		Aut-chr	1/8			
		
	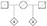	X-chr	1/8	1/2	1/4	1/4

Paternal grandparenthood		Aut-chr	1/8			
		
	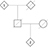	X-chr	1/4	0	0	0

Maternal grandparenthood		Aut-chr	1/8			
		
	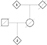	X-chr	1/8	1/2	1/4	1/4

Paternal avuncular		Aut-chr	1/8			
		
	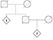	X-chr	1/8	0	0	1/4

Maternal avuncular		Aut-chr	1/8			
		
	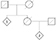	X-chr	3/16	1/4	3/8	1/8

* *k*_*i*_, probability of sharing *i* pairs of IBD autosomal alleles; *x*_*i*_, probability of sharing *i* pairs of IBD X-chromosomal alleles.

### Parenthood Testing

The X-chromosomal markers can be used to complement autosomal information when inconclusive or weak statistical results are achieved in standard parenthood testing where the alternative hypothesis is the individuals being unrelated. This can be due to the poor quality or low quantity of DNA in degraded samples, resulting in few analyzed markers or to other, more complex, situations where few Mendelian incompatibilities are found.

Compared with autosomes, X-chromosomal markers provide greater statistical power in trios, in paternity duos with daughters, and in maternity duos with sons. The X-chromosomal markers are not informative in paternity cases with sons, and for mother/daughter duos, the same statistical power is obtained for autosomal and X-chromosomal transmission.

When few Mendelian incompatibilities are found, this can be due to the alleged parent of the child being related to the true parent. A relatively common situation is the alleged father being either a full brother or the father of the true father of the child, in which case the probability of the alleged father and child sharing a pair of IBD alleles is 50%. In a paternity testing with a daughter, if the alleged father is a brother of the real one, the probability of uncle–niece sharing a pair of IBD X-alleles is also 50%. In all the other cases, this probability is null. Indeed, the analysis of X-chromosomal markers can be an efficient approach for excluding close relatives of the real father, unknowingly presented in a standard paternity case (Gomes et al., 2012).

### Beyond Parenthood

In some cases, the alleged parent is not available for analysis, and sibship, or grandparenthood problems may emerge. In some of these cases, X-chromosomal markers can provide invaluable information, stronger than the one provided by autosomes. The most striking examples are those where the sharing of a pair of IBD X-alleles is mandatory. This occurs when the paternity of a daughter is questioned, being the alleged father unavailable for analysis, contrarily to his (unquestioned) mother or daughter. In both cases, the sharing of IBS alleles between analyzed females is mandatory for all the markers, unless mutation occurs. In these cases, the reached statistical power is the same for a paternity testing with autosomes when the alleged father is directly analyzed whether the mother of the child is available for analysis or not.

Another illustrating example is the kinship problem where the hypotheses are “full sisters” versus “unrelated.” Considering X-chromosomal transmission and the main hypothesis, females share either two or one pair of IBD X-alleles with the same probability: 50%. Assuming autosomal transmission, they may not share IBD alleles (with 25% of chance), such as occurs assuming they are unrelated (with 100% of chance). It is then expected that X-chromosomal markers provide stronger results than autosomes. This occurs in all the kinships where the transmission of the X chromosome is not interrupted due to its obligatory transmission between father and daughter, which allows the skipping of one meiosis.

### Incest Cases

In some cases, the high number of homozygosities shown by a child (e.g., in a paternity testing with alleged father excluded) may raise the suspicion of an incestuous situation. This may, under some circumstances, configure a crime (mother under age or with intellectual disability, for example). In the case of a daughter, X-chromosomal analyses may provide important insights even without analyzing the alleged father. If the father of the daughter is also the father of the mother and, in the absence of mutation, either the child is homozygous (for one allele present in the mother) or is heterozygous for the same alleles of the mother. In the case of autosomal transmission, three alleles can be seen in mother/daughter pair, as for the case of the parents being unrelated.

The hypotheses of the father of the child being either the father or the full brother of the mother are theoretically indistinguishable when considering unlinked autosomal markers. Contrastingly, in the case of daughters, X-chromosomal markers can provide insights allowing the different weighing of the two hypotheses (Pinto et al., 2011a).

### Distinguishing Pedigrees Belonging to the Same Autosomal Kinship Class

Pedigrees are theoretically indistinguishable, considering unlinked markers, whenever they have the same IBD partitions (Pinto et al., 2010). This is the case of the second-degree relatives: avuncular, half-siblings and grandparent–grandchild, as the probability of individuals sharing two pairs of IBD alleles is null, while the probability of sharing one pair of IBD autosomal alleles is equal to the probability of sharing none (50%) – see Table 4. Nevertheless, the analysis of X-chromosomal markers can provide differential weighing favoring one of the alternative hypotheses (Pinto et al., 2011a). For example, when a pair of females is analyzed, maternal and paternal aunt/niece can be distinguished from, respectively, maternal and paternal half-sisters and grandmother–granddaughter, which are not distinguishable among them even when considering X-chromosomal markers. In all the cases, females cannot share two pairs of IBD alleles, but a pair of maternal aunt/niece shares one pair of IBD alleles with a probability equal to 75%, while for both maternal half-sisters and grandmother–granddaughter pairs, this probability reduces to 50%. On the other, if both pairs of paternal half-sisters and grandmother–granddaughter have to share one pair of IBD alleles, this probability drops from 100 to 50% in the case of paternal aunt/niece. Different IBD probabilities will result in different weighing of the evidence, depending on the genotypic observations.

## Segregation Studies: Current Data and Missing Data

The high power of discrimination that characterizes STRs and makes them desirable genetic markers compared to SNPs or INDELs, particularly in human identification analysis (such as kinship testing), is due to their higher mutation rate. An STR is, by definition, a tandemly arrayed repetition of a DNA fragment of one to six base pairs. There is general consensus that these are created by random mutations (Levinson and Gutman, 1987; Schlötterer, 2000). Generally, STRs with four base pairs motifs are plentiful and more stable than two or three nucleotide repeats; hence, they have been favored when designing the commercially available forensic kits (Pereira and Gusmão, 2016). Motifs with two or three base pairs are less stable and have a higher propensity for stutter during PCR, and STRs with more base pairs are less frequent. When a somatic mutation occurs, it affects only cell lines of the individual where it occurred. However, when a mutation occurs in the germ line, it has the potential of being passed on to the offspring and resulting in different parental and filial alleles. Mutation rates vary between types of polymorphisms and also on inherent individual characteristics such as sex and age (Brinkmann et al., 1998; Nachman and Crowell, 2000).

Polymerase template slippage is thought to be the primary mutational mechanism leading to changes in STR length (Schlötterer and Tautz, 1992; Strand et al., 1993), and mutations involving the loss or gain of one repeat are assumed to be preponderant over mutations involving the loss or gain of multiple repeats. Slippage occurs during DNA replication when the two DNA strands come apart. When misalignment occurs out of register the repeat number of the STR product will be different. The currently accepted mutational model, also known as the stepwise mutation model (SMM) (Ohta and Kimura, 1973) occurring as a result of DNA replication slippage, includes mutational forces working in opposite directions: polymerase template slippage and point mutations; the latter reduce the length of STRs due to the breakage of the original segment creating two new shorter segments. Studies have shown that the longer the allele length, the higher is the frequency of these events. It has also been reported that longer alleles tend to mutate to shorter alleles and vice versa, while intermediate-sized alleles have approximately the same tendency to shorten or lengthen (Primmer et al., 1996; Brinkmann et al., 1998; Xu et al., 2000; Antão-Sousa et al., 2019).

In forensic casework context, the estimation of mutation rates is crucial for the analysis, interpretation, and quantification of experimental data and for the proper quantification of LRs. In such scenarios, the detection of mutation(s) has practical consequences in the interpretation of the genetic profiles. Some studies have addressed this by analyzing different familial configurations, familial duos, mother–son, mother–daughter, and father–daughter, and familial trios, father–mother–daughter (e.g., Jin et al., 2016; Burgos et al., 2019; García et al., 2019). Supplementary Table 3 presents the most updated information on mutation rates per marker and per familial configuration for the most commonly used X-STRs. To date, not much research on the mutation rates of the most commonly used X-STRs has been given, and therefore, data collection and analyses are still lacking. Perhaps one of the limitations in the estimation of mutation rates of STRs, in general, is the use of the (most frequently used) method for mutation estimates based on direct pedigree analysis. This means that mutated alleles are identified straightforward by the observation of allele transmissions in parent–child requiring a large amount of data to reliably estimate allele mutation rates. Having access to a high number of specific constellations of families may be a drawback to the (accurate) estimation of mutation rates of X-STRs.

## Discussion

### Factors Underlying the Relative Stagnation in X Chromosome Forensic Research

After an initial boom, forensic research interest on X chromosome markers has witnessed a decline as judged by the number of relevant publications: 2000 (6), 2001 (7), 2002 (11), 2003 (18), 2004 (27), 2005 (25), 2006 (40), 2007 (35), 2008 (42), 2009 (43), 2010 (19), 2011 (41), 2012 (26), 2013 (22), 2014 (18), 2015 (15), 2016 (22), 2017 (31); 2018 (16), and 2019 (18) [search results obtained using Scopus database^5^ and the following criteria [ALL (dxs^∗^) AND ALL (forensic)] AND PUBYEAR > 1999 AND PUBYEAR < 2020 on 30/04/2020]. In the beginning of the early 2000s, only a scarce number of X chromosome STRs and a very limited number of human population groups were characterized for forensic genetic applications. Data focusing on the assessment of X-linked polymorphisms for forensic and kinship genetic studies were an impending demand which created a gap in these fields producing sufficient ground for the interest in X chromosome markers and, in particular, X-STRs. Consequently, an increase of studies in 2003 until 2011 (with exception of the year 2010) can be noted. After this year, fluctuations are mostly toward a reduction of X-STR studies (except for 2017).

This implies that the practical forensic use of X chromosome is well below its potential and – what is most concerning – is that its use may be unsupported by research data and based on inadequately validated technical means and theoretically reduced or even incorrect analytical approaches. Enabling corrective actions demands therefore the identification of the causes of this slowing down of the forensically inclined research on X genetic markers. This fact has no parallel on the other sexual chromosome counterpart, the Y, to which a lot of attention is devoted, for example, by the STRbase (National Institute of Standards, and Technology [Nist], 2020) and has as well a very active dedicated site^6^, YHRD (2020), in contrast to the ChrX-STR.org 2, as mentioned previously (see text footnote 2).

In this section, we will analyze the putative change counteracting the loss of interest and analyzing the presumed reasons or factors justifying this situation, which, from our point of view, can be classified into four broad categories: (a) theoretical and/or analytical, (b) technical, (c) statistical, and (d) medical/ethical, to be detailed below.

### Theoretical and Analytical Difficulties

The main obstacle to the correct use of X chromosome in forensics lies in the hybrid nature of its formal genetic model of inheritance, common to most mammals, with very few exceptions (Cortez et al., 2014; Matveevsky et al., 2017). Indeed, as presented in the section “Introduction,” this chromosome harbors two distinct modes of transmission: the diploid, autosomal style (corresponding to the so-called pseudoautosomal regions), two in humans, PAR 1 and PAR 2 (Flaquer et al., 2009) and the sex-linked haplodiploid (for the rest of the chromosome, known as X-specific), which, due to the single copy in males, does not recombine.

When addressing X-chromosome markers, we are referring to the X-specific located ones. Therefore, only these will be analyzed (although some confusions do sometimes arise and quite often the status of X specificity may be doubtful – see below the technical section).

Even so, the formal genetic model of transmission and the consequences at the level of population genetics seem to be poorly understood by the forensic community, as judged by a recent analysis of the literature (Ferragut et al., 2019). It was shown that in 60% of 52 analyzed publications, forensic parameters were computed as for autosomal markers, and the analysis of associations between alleles from distinct loci (LD) was generally deficient or erroneous. In fact, linkage and LD concepts, particularly important for the X chromosome since all markers are located on the same chromosome, are often a source of confusion and generally lead to misinterpretation or even non-consideration of LD results in many genetic studies. Most studies using X-STRs correctly test for the presence of significant association among pairs of loci (LD) but fail to estimate haplotype frequencies and probability calculations, accordingly, when significant association is found among markers as loci must be analyzed together and not as individual markers in such cases. In 2017, recommendations were provided by the DNA commission of the ISFG addressing exactly the issues behind the concepts of linkage and LD in cases of kinship testing using X-STRs and emphasizing that “Haplotype frequencies should be used for likelihood calculations when LD exists” (Tillmar et al., 2017).

Similar issues have also arisen with the assessment of conformity with Hardy–Weinberg equilibrium expectations. Quite symptomatically, the ChrX-STR.org 2 website (see text footnote 2, accessed on 02/05/2020) has posted: “Based on the review of December 2018, it has been decided in cooperation with the X working group to remove the PI calculation from this website.”

From an applicable point of view, one can add that one of the additional problems to justify the decrease of interest in X chromosome markers could be due to the low number of identification cases that request X-STR markers. Perhaps the troubles behind the implementation of a new system (financial cost and human resource training) which has a much more complex type of analysis when compared to the Y chromosome, for example, may not justify the need for the use of this system.

### Technical Problems

Besides the genotyping problems, which may be transversal to all markers, irrespectively of the mode of transmission, sex chromosomes pose special difficulties due to their complex evolutionary history. In fact, apart from the PAR regions, X and Y chromosomes still keep substantial extensions of homologous regions, which obstruct the safe establishment of specificity for a marker, as well as its primers in case of PCR-based techniques. Particularly for recently X/Y transposed regions, this may constitute an (nearly) insurmountable obstacle (Lopes et al., 2004) as well as the dynamic state of the pseudoautosomal moving boundaries (Otto et al., 2011).

### Statistical Issues

Most of the statistical problems (both at the descriptive level – parameter estimation level or hypothesis testing design or evidence quantitative evaluation) stem out of the theoretical flaws discussed above. Nonetheless, some are specifically empirical and are related to the haplodiploid specificity of the X chromosome: different sampling and estimation methods are required for each sex. Indeed, while haplotype frequencies can be estimated by simple counting in males, in females, they have to be inferred. Needless to say, simple haplotype frequency estimation requires prohibitively large sample sizes, growing exponentially with the number of loci involved (Amorim and Pinto, 2018).

### Medical/Ethical Questions

To begin with, it must be highlighted that the very genotyping of sex chromosome markers for forensic purposes may represent a violation of some of the established recommendations and rules on the exclusion of any markers that can reveal physical traits [e.g., European Council Resolution of 25 June 2001 on the exchange of DNA analysis results (2001/C 187/01)]. Furthermore, gender and sex are always sensitive, and sometimes conflicting, categories in or for some individuals.

The evolutionary dynamics of sex chromosomes introduces also undesirable clinical and ethical problems. In fact, sex chromosomes are the Achilles’ heel of male meiosis (Kauppi et al., 2012). A non-negligible proportion (1/448 live births) of the human population carries some sort of chromosomal aberration and, for example, one of the aneuploidies, Klinefelter syndrome, has an incidence of ∼1/500 male live births) (Nielsen and Wohlert, 1990). The consequences for forensic practice are ethically troublesome: discordance of external sex from X chromosome typing and unwilling disclosure of a clinical condition. In addition to X-chromosomal changes, several X-STR markers, that were or are still in use, have been linked to medical conditions. The HumARA is linked to spinal and bulbar muscular dystrophy (SBMA) as well as to other health risks (Szibor et al., 2005). Another example is the possible LD between the STR alleles at HPRTB locus to the X-linked recessive disorder Lesch–Nyhan syndrome (caused by molecular defects within the HPRT gene) (Mansfield et al., 1993). Some data have shown that inheritance of two polymorphic tandem repeats, one being the HPRTB locus (mapped within intron 3 of the HPRT gene), could be used to establish linkage to the disease (Mansfield et al., 1993).

The X chromosome has had an interesting journey in the last two decades in the research fields of forensic and population genetics by providing new (population) data and aiding in the clarification of several issues, namely, in kinship testing. Its particular properties of inheritance (recombination on the female side and haploid state on the male side) have allowed this chromosome a role that cannot be accomplished by the autosomes neither by its counterpart, the Y chromosome. After an initial bloom of publications, several multiplex developments, workshops at international meetings, creation of an X-STR database, the interest in X-chromosomal markers is gradually fading. Analytical and statistical issues may be the major underlined motivations to the lack of interest in addition to a lower demand of X-STR-based identification cases.

Considerable effort has already been put in X-STRs, namely, (i) the generation of allelic and haplotypic frequency databases that include a fair enough number of geographically different located populations; (ii) several in-house multiplexes containing a large number of highly polymorphic markers as well as a sound established commercial kit; and (iii) relevant number of studies addressing and recommending solutions for the main issues surrounding X-STR kinship-based testing. Therefore, this effort should not be lost and move toward the revival of the standing position of X chromosome markers in forensic genetics.

## Author Contributions

IG drafted the manuscript scheme and, in addition, all authors have made a substantial, direct, and intellectual contribution to the work, and approved the final version for publication.

## Conflict of Interest

The authors declare that the research was conducted in the absence of any commercial or financial relationships that could be construed as a potential conflict of interest.

## Footnotes

1 http://famlink.se/Databases/

2 http://www.chrx-str.org/

3 https://strider.online/

4 www.isfg.org

5 http://www.scopus.com

6 https://yhrd.org/

## References

[B1] Amorim A. Pereira L. (2005). Pros and cons in the use of SNPs in forensic kinship investigation: a comparative analysis with STRs. *Forensic Sci. Int.* 150 17–21. 10.1016/j.forsciint.2004.06.018 15837005

[B2] Amorim A. Pinto N. (2018). Big data in forensic genetics. *Forensic Sci Int Genet.* 37 102–105. 10.1016/j.fsigen.2018.08.001 30142461

[B3] Antão-Sousa S. Amorim A. Gusmão L. Pinto N. (2019). Mutation in Y STRs: Repeat motif gains vs. losses. *Forensic Science International: Genetics Supplement Series* 7 240–242. 10.1016/j.fsigss.2019.09.092

[B4] Bär W. Brinkmann B. Budowle B. Carracedo A. Gill P. Lincoln P. (1997). DNA recommendations. Further report of the DNA Commission of the ISFH regarding the use of short tandem repeat systems. International Society for Forensic Haemogenetics. *Int J Legal Med* 110 175–176. 10.1007/s004140050061 9274938

[B5] Bini C. Di Nunzio C. Aneli S. Sarno S. Alù M. Carnevali E. (2019). Analysis of recombination and mutation events for 12 X-Chr STR loci: A collaborative family study of the Italian Speaking Working Group Ge.F.I. *Forensic Science International: Genetics Supplement Series* 7 398–400. 10.1016/j.fsigss.2019.10.027

[B6] Brinkmann B. Klintschar M. Neuhuber F. Hühne J. Rolf B. (1998). Mutation rate in human microsatellites: influence of the structure and length of the tandem repeat. *The American Journal of Human Genetics* 62 1408–1415. 10.1086/301869 9585597 PMC1377148

[B7] Budowle B. van Daal A. (2008). Forensically relevant SNP classes. *Biotechniques* 44 603–608,610. 10.2144/000112806 18474034

[B8] Burgos G. Posada Y. Florez-Misas A. Ávila C. Ibarra A. (2019). An update of STR mutation rates from paternity tests analyzed in a 14 year period (2005–2018) at IdentiGEN lab, Universidad de Antioquia, Colombia. *Forensic Science International: Genetics Supplement Series* 7 530–531. 10.1016/j.fsigss.2019.10.078

[B9] Butler J. M. Coble M. C. Vallone P. M. (2007). STRs vs. SNPs: thoughts on the future of forensic DNA testing. *Forensic sci med pathol* 3 200–205. 10.1007/s12024-007-0018-1 25869164

[B10] Carvalho R. Pinheiro M. F. (2011). Study of DXS9895 and DXS7130: Population data from North of Portugal. *J Forensic Leg Med.* 18 21–22. 10.1016/j.jflm.2010.11.010 21216375

[B11] Chakraborty R. Stivers D. N. Su B. Zhong Y. Budowle B. (1999). The utility of short tandem repeat loci beyond human identification: implications for development of new DNA typing systems. *Electrophoresis* 20 1682–1696. 10.1002/(SICI)1522-2683(19990101)20:8<1682::AID-ELPS1682<3.0.CO;2-Z 10435432

[B12] ChrX-Str.org 2.0. (2020). *ChrX-STR.org 2.0.* http://www.chrx-str.org/ (accessed May 15, 2020)

[B13] Cortez D. Marin R. Toledo-Flores D. Froidevaux L. Liechti A. Waters P. D. (2014). Origins and functional evolution of Y chromosomes across mammals. *Nature* 508 488–493. 10.1038/nature13151 24759410

[B14] Deng C. Song F. Li J. Hou Y. Luo H. (2017). Multiplex PCR for 19 X-chromosomal STRs in Chinese population. *Forensic Science International: Genetics Supplement Series* 6 e24–e26. 10.1016/j.fsigss.2017.09.016

[B15] Diegoli T. M. (2015). Forensic typing of short tandem repeat markers on the X and Y chromosomes. *Forensic Sci. Int. Genet.* 18 140–151. 10.1016/j.fsigen.2015.03.013 25934544

[B16] Diegoli T. M. Rohde H. Borowski S. Krawczak M. Coble M. D. Nothnagel M. (2016). Genetic mapping of 15 human X-chromosomal forensic short tandem repeat (STR) loci by means of multi-core parallelization. *Forensic Sci. Int. Genet.* 25 39–44. 10.1016/j.fsigen.2016.07.004 27497644

[B17] Edelmann J. Kohl M. Dressler J. Hoffmann A. (2016). X-chromosomal 21-indel marker panel in German and Baltic populations. *Int. J. Legal. Med.* 130 357–360. 10.1007/s00414-015-1221-3 26164591

[B18] Edelmann J. Lessig R. Willenberg A. Wildgrube R. Hering S. Szibor R. (2006). Forensic validation of the X-chromosomal STR-markers GATA165B12, GATA164A09, DXS9908 and DXS7127 in German population. *International Congress Series* 1288 298–300. 10.1016/j.ics.2005.09.022

[B19] Edwards A. Civitello A. Hammond H. A. Caskey C. T. (1991). DNA Typing and Genetic Mapping With Trimeric and Tetrameric Tandem Repeats. *Am. J. Hum. Genet.* 49 746–756. 1897522 PMC1683171

[B20] Edwards A. Hammond H. A. Jin L. Caskey C. T. Chakraborty R. (1992). Genetic Variation at Five Trimeric and Tetrameric Tandem Repeat Loci in Four Human Population Groups. *Genomics* 12 241–253. 10.1016/0888-7543(92)90371-x 1740333

[B21] Egeland T. Pinto N. Vigeland M. D. (2014). A general approach to power calculation for relationship testing. *Forensic Sci. Int. Genet.* 9 186–190. 10.1016/j.fsigen.2013.05.001 23810238

[B22] Elakkary S. Hoffmeister-Ullerich S. Schulze C. Seif E. Sheta A. Hering S. (2014). Genetic polymorphisms of twelve X-STRs of the investigator Argus X-12 kit and additional six X-STR centromere region loci in an Egyptian population sample. *Forensic Sci Int Genet.* 11 26–30. 10.1016/j.fsigen.2014.02.007 24632058

[B23] Fan G. Ye Y. Luo H. Hou Y. (2015). Screening of Multi-InDel markers on X-chromosome for forensic purpose. *Forensic Science International: Genetics Supplement Series* 5 e42–e44. 10.1016/j.fsigss.2015.09.017

[B24] Fan G. Y. Ye Y. Hou Y. P. (2016). Detecting a hierarchical genetic population structure via Multi-InDel markers on the X chromosome. *Scientific Reports* 6 32178. 10.1038/srep32178 PMC4989243 27535707

[B25] Ferragut J. F. Pinto N. Amorim A. Picornell A. (2019). Improving publication quality and the importance of Post Publication Peer Review: The illustrating example of X chromosome analysis and calculation of forensic parameters. *Forensic Sci Int Genet.* 38 e5–e7. 10.1016/j.fsigen.2018.11.006 30455113

[B26] Flaquer A. Fischer C. Wienker T. F. (2009). A new sex-specific genetic map of the human pseudoautosomal regions (PAR1 and PAR2). *Hum Hered.* 68 192–200. 10.1159/000224639 19521101

[B27] Freitas N. S. Resque R. L. Ribeiro-Rodrigues E. M. Guerreiro J. F. Santos N. P. Ribeiro-Dos-Santos A. (2010). X-linked insertion/deletion polymorphisms: forensic applications of a 33-markers panel. *Int J Legal Med.* 124 589–593. 10.1007/s00414-010-0441-9 20354713

[B28] Fukuta M. Gaballah M. Takada K. Miyazaki H. Kato H. Aoki Y. (2019). Genetic polymorphism of 27 X-chromosomal short tandem repeats in an Egyptian population. *Legal Medicine* 37 64–66. 10.1016/j.legalmed.2019.01.009 30711876

[B29] Gao H. Wang C. Zhang R. Wu H. SunS Xiao D. (2019). Application of CPI cutoff value based on parentage testing of duos and trios typed by four autosomal kits. *PLoS ONE* 14:e0225174. 10.1371/journal.pone.0225174 31721797 PMC6853303

[B30] García M. G. Catanesi C. I. Penacino G. A. Gusmão L. Pinto N. (2019). X-chromosome data for 12 STRs: Towards an Argentinian database of forensic haplotype frequencies. *Forensic Science International: Genetics* 41 e8–e13. 10.1016/j.fsigen.2019.04.005 31085140

[B31] Gettings K. B. Aponte R. A. Vallone P. M. Butler J. M. (2015). Current knowledge and future issues. *Forensic Sci Int Genet* 18 118–130. 10.1016/j.fsigen.2015.06.005 26197946

[B32] Gill P. Brenner C. Brinkmann B. Budowle B. Carracedo A. Jobling M. A. (2001). DNA Commission of the International Society of Forensic Genetics: recommendations on forensic analysis using Y-chromosome STRs. *Forensic Sci. Int.* 124 5–10. 10.1016/s0379-0738(01)00498-4 11741752

[B33] Gjertson D. W. Brenner C. H. Baur M. P. Carracedo A. Guidet F. Luque J. A. (2007). ISFG: recommendations on biostatistics in paternity testing. *Forensic Sci. Int. Genet* 1 223–231. 10.1016/j.fsigen.2007.06.006 19083766

[B34] Gomes C. Magalhães M. Alves C. Amorim A. Pinto N. Gusmão L. (2012). Comparative evaluation of alternative batteries of genetic markers to complement autosomal STRs in kinship investigations: autosomal indels vs. X-chromosome STRs. *Int. J. Legal Med.* 126 917–921. 10.1007/s00414-012-0768-5 22940765

[B35] Gomes I. Brehm A. Gusmão L. Schneider P. M. (2016). New Sequence Variants Detected at DXS10148, DXS10074 and DXS10134 Loci. *Forensic Sci Int Genet* 20 112–116. 10.1016/j.fsigen.2015.10.005 26590332

[B36] Gomes I. Pereira P. J. P. Harms S. Oliveira A. M. Schneider P. M. Brehm A. (2017). Genetic characterization of Guinea-Bissau using a 12 X-chromosomal STR system: Inferences from a multiethnic population. *Forensic Sci Int Genet.* 31 89–94. 10.1016/j.fsigen.2017.08.016 28858674

[B37] Gomes I. Pereira R. Mayr W. R. Amorim A. Carracedo A. Gusmão L. (2009). Evaluation of DXS9902, DXS7132, DXS6809, DXS7133, and DXS7423 in humans and chimpanzees: sequence variation, repeat structure, and nomenclature. *Int. J. Legal Med.* 123 403–412. 10.1007/s00414-009-0357-4 19536558

[B38] Gomes I. Prinz M. Pereira R. Bieschke E. Amorim A. Carracedo A. (2008). Sequence variation at three X chromosomal short tandem repeats in Caucasian and African populations. *Forensic Science International: Genetics Supplement Series* 1 147–149. 10.1016/j.fsigss.2007.10.097

[B39] Gunter C. (2005). Genome Biology: She Moves in Mysterious Ways. *Nature* 434 279–280. 10.1038/434279a 15772630

[B40] Gusmão L. Butler J. M. Carracedo A. Gill P. Kayser M. Mayr W. R. (2006). International Society of Forensic Genetics. DNA Commission of the International Society of Forensic Genetics (ISFG): an update of the recommendations on the use of Y-STRs in forensic analysis. *Int J Legal Med* 120 191–200. 10.1007/s00414-005-0026-1 16998969

[B41] Gusmão L. Butler J. M. Linacre A. Parson W. Roewer L. Schneider P. M. (2017). Revised guidelines for the publication of genetic population data. *Forensic Sci Int Genet.* 30 160–163. 10.1016/j.fsigen.2017.06.007 28673498

[B42] Gusmão L. González-Neira A. Alves C. Lareu M. Costa S. Amorim A. (2002). Chimpanzee homologous of human Y specific STRs: A comparative study and a proposal for nomenclature. *Forensic Sci. Int.* 126 129–136. 10.1016/s0379-0738(02)00046-4 12084489

[B43] Gusmão L. Sánchez-Diz P. Alves C. Gomes I. Zarrabeitia M. T. Abovich M. (2008). A GEP-ISFG collaborative study on the optimization of an X-STR decaplex: data on 15 Iberian and Latin American populations. *Int. J. Legal Med.* 123 227–234. 19082839 10.1007/s00414-008-0309-4

[B44] Gusmão L. Sánchez-Diz P. Alves C. Gomes I. Zarrabeitia M. T. Abovich M. (2009). A GEP-ISFG collaborative study on the optimization of an X-STR decaplex: data on 15 Iberian and Latin American populations. *Int. J. Legal Medicine* 123 227–234. 10.1007/s00414-008-0309-4 19082839

[B45] Haldane J. B. S. (1919). The combination of linkage values, and the calculation of distances between the loci of linked factors. *J. Genet.* 8 299–309.

[B46] Hearne C. M. Todd J. A. (1991). Tetranucleotide repeat polymorphism at the HPRT locus. *Nucleic Acids Res.* 19 5450 10.1093/nar/19.19.5450 PMC328931 1923839

[B47] Hedrick P. W. (1987). Gametic disequilibrium measures: proceed with caution. *Genetics* 117 331–341. 3666445 10.1093/genetics/117.2.331 PMC1203208

[B48] Hwa H. L. Chung W. C. Chen P. L. Lind C. L. Lie H. Y. Yin H. I. (2018). A 1204-single nucleotide polymorphism and insertion-deletion polymorphism panel for massively parallel sequencing analysis of DNA mixtures. *Forensic Sci Int Genet.* 32 94–101. 10.1016/j.fsigen.2017.11.002 29128546

[B49] Hwa H. L. Wu M. Y. Lin C. P. Hsieh W. H. Yin H. I. Lee T. T. (2019). A single nucleotide polymorphism panel for individual identification and ancestry assignment in Caucasians and four East and Southeast Asian populations using a machine learning classifier. *Forensic Sci Med Pathol.* 15 67–74. 10.1007/s12024-018-0071-y 30649693

[B50] Jacquard A. (1974). *The genetic structure of populations; in Biomathematics*, Vol. 5 Berlin: Springer-Verlag, 102–107.

[B51] Jäger A. C. Alvarez M. L. Davis C. P. Guzmán E. Han Y. Way L. (2017). Developmental validation of the MiSeq FGx Forensic Genomics System for Targeted Next Generation Sequencing in Forensic DNA Casework and Database Laboratories. *Forensic Sci Int Genet.* 28 52–70. 10.1016/j.fsigen.2017.01.011 28171784

[B52] Jin B. Su Q. Luo H. Li Y. Wu J. Yan J. (2016). Mutational analysis of 33 autosomal short tandem repeat (STR) loci in southwest Chinese Han population based on trio parentage testing. *Forensic Science International: Genetics* 23 86–90. 10.1016/j.fsigen.2016.03.009 27045978

[B53] Kauppi L. Jasin M. Keeney S. (2012). The tricky path to recombining X and Y chromosomes in meiosis. *Ann. N. Y. Acad. Sci.* 1267 18–23. 10.1111/j.1749-6632.2012.06593.x 22954211 PMC3631422

[B54] Kling D. Dell’Amico B. Tillmar A. O. (2015a). FamLinkX - implementation of a general model for likelihood computations for X-chromosomal marker data. *Forensic Sci. Int. Genet.* 17 1–7. 10.1016/j.fsigen.2015.02.007 25771099

[B55] Kling D. Tillmar A. Egeland T. Mostad P. (2015b). A general model for likelihood computations of genetic marker data accounting for linkage, linkage disequilibrium, and mutations. *Int. J. Legal Med.* 129 943–954. 10.1007/s00414-014-1117-7 25425094

[B56] Kohn M. Kehrer-Sawatzki H. Vogel W. Graves J. A. Hameister H. (2004). Wide genome comparisons reveal the origins of the human X chromosome. *Trends Genet.* 20 598–603. 10.1016/j.tig.2004.09.008 15522454

[B57] Kosambi D. D. (1944). The estimation of map distances from recombination values. *Ann. Eugen.* 12 172–175. 10.1111/j.1469-1809.1943.tb02321.x

[B58] Koyama H. Iwasa M. Tsuchimochi T. Maeno Y. Isobe I. Seko-Nakamura Y. (2002). Y-STR haplotype data and allele frequency of the DXS10011 locus in a Japanese population sample. *Forensic Sci. Int.* 125 273–276. 10.1016/s0379-0738(01)00649-1 11909676

[B59] Krawczak M. (2007). Kinship testing with X-chromosomal markers: mathematical and statistical issues. *Forensic Sci. Int. Genet.* 1 111–114. 10.1016/j.fsigen.2007.01.014 19083739

[B60] Lahn B. T. Page D. C. (1999). Four evolutionary strata on the human X chromosome. *Science* (1999). 286 964–967. 10.1126/science.286.5441.964 10542153

[B61] Lander E. S. Linton L. M. Birren B. Nusbaum C. Zody M. C. Baldwin J. (2001). Initial sequencing and analysis of the human genome. *Nature* 409 860–921. 10.1038/35057062 11237011

[B62] Lazaruk K. Wallin J. Holt C. Nguyen T. Walsh P. S. (2001). Sequence variation in humans and other primates at six short tandem repeat loci used in forensic identity testing. *Forensic Sci. Int.* 119 1–10. 10.1016/s0379-0738(00)00388-1 11348787

[B63] Levinson G. Gutman G. A. (1987). Slipped-strand mispairing: a major mechanism for DNA sequence evolution. *Mol. Biol. Evol.* 4 203–221. 10.1093/oxfordjournals.molbev.a040442 3328815

[B64] Li L. Li C. Zhang S. Zhao S. Liu Y. Lin Y. (2010). Analysis of 14 highly informative SNP markers on X chromosome by TaqMan SNP genotyping assay. *Forensic Sci Int Genet.* 4 e145–e148. 10.1016/j.fsigen.2010.04.004 20493796

[B65] Li J. Deng C. Luo H. Song F. Hou Y. (2017). *Analyzing an off Forensic Science International: Genetics Supplement Series* 6 e92–e93.

[B66] Lin L. Li J. Hu Y. Wang H. Marah F. A. Moseray M. (2020). Genetic characterization of 19 X-STRs in Sierra Leone population from Freetown. *Int. J. Legal Med.* 34, 1659–1661. 10.1007/s00414-019-02243-6 31897669

[B67] Liu Q.-L. Wang J.-Z. Quan L. Zhao H. Wu Y.-D. Huang X.-L. (2013). Allele and Haplotype Diversity of 26 X-STR Loci in Four Nationality Populations from China. *PLoS ONE* 8:e65570. 10.1371/journal.pone.0065570 23805185 PMC3689794

[B68] Lopes A. M. Calafell F. Amorim A. (2004). Microsatellite variation and evolutionary history of PCDHX/Y gene pair within the Xq21.3*/Yp*11.2 hominid-specific homology block. *Mol Biol Evol.* 21 2092–2101. 10.1093/molbev/msh218 15297598

[B69] Mansfield E. S. Blasband A. Kronick M. N. Wrabetz L. Kaplan P. Rappaport E. (1993). Fluorescent approaches to diagnosis of Lesch-Nyhan syndrome and quantitative analysis of carrier status. *Mol. Cell. Probes* 7 311–324. 10.1006/mcpr.1993.1045 8232348

[B70] Matveevsky S. Kolomiets O. Bogdanov A. Hakhverdyan M. Bakloushinskaya I. (2017). Chromosomal Evolution in Mole Voles Ellobius (Cricetidae, Rodentia): Bizarre Sex Chromosomes, Variable Autosomes and Meiosis. *Genes (Basel).* 8 306. 10.3390/genes8110306 29099806 PMC5704219

[B71] Medina-Acosta E. (2011). Evidence of partial and weak gametic disequilibrium across clusters of pericentromeric short tandem repeats loci on human X chromosome: proceed with caution in forensic genetics. *Forensic Sci Int Genet.* 5 545–547. 10.1016/j.fsigen.2009.12.002 20457061

[B72] Nachman M. W. Crowell S. L. (2000). Estimate of the mutation rate per nucleotide in humans. *Genetics* 156 297–304. 10978293 10.1093/genetics/156.1.297 PMC1461236

[B73] National Institute of Standards, and Technology [Nist]. (2020). *Standard Reference Database.* Avaliable at: https://strbase.nist.gov/ (accessed May 10, 2020).

[B74] Nielsen J. Wohlert M. (1990). Sex chromosome abnormalities found among 34,910 newborn children: results from a 13-year incidence study in Arhus. *Denmark. Birth Defects Original Article Series* 26 209–223. 10.1007/BF01213097 2090319

[B75] Nishi T. Fukui K. Iwadate K. (2020). Genetic Polymorphism Analyses of Three Novel X Chromosomal Short Tandem Repeat Loci in the Xp22.3 Region. *Legal Medicine* 45 101709. 10.1016/j.legalmed.2020.101709 32371301

[B76] Nothnagel M. Szibor R. Vollrath O. Augustin C. Edelmann J. Geppert M. (2012). Collaborative genetic mapping of 12 forensic short tandem repeat (STR) loci on the human X chromosome. *Forensic Sci. Int. Genet.* 6 778–784. 10.1016/j.fsigen.2012.02.015 22459949

[B77] Ohta T. Kimura M. (1973). A model of mutation appropriate to estimate the number of electrophoretically detectable alleles in a finite population. *Genet. Res.* 22 201–204. 10.1073/pnas.75.6.2868 4777279

[B78] Oki T. Hayashi T. Ota M. Asamura H. (2012). Development of multiplex assay with 16 SNPs on X chromosome for degraded samples. *Leg Med (Tokyo)* 14 11–16. 10.1016/j.legalmed.2011.10.001 22177906

[B79] Olaisen B. Bär W. Brinkmann B. Budowle B. Carracedo A. Gill P. (1998). DNA recommendations 1997 of the International Society for Forensic Genetics. *Vox Sang* 74 61–63. 9481867

[B80] Otto S. Pannell J. R. Peichel C. L. Ashman T. L. Charlesworth D. Chippindale A. K. (2011). About PAR: the distinct evolutionary dynamics of the pseudoautosomal region. *Trends Genet.* 27 358–367. 10.1016/j.tig.2011.05.001 21962971

[B81] Parson W. Roewer L. (2010). Publication of Population Data of Linearly Inherited DNA Markers in the International Journal of Legal Medicine. *Int J Legal Med.* 124 505–509. 10.1007/s00414-010-0492-y 20652581

[B82] Pereira R. Alves C. Aler M. Amorim A. Arévalo C. Betancor E. (2018). A GHEP-ISFG collaborative study on the genetic variation of 38 autosomal indels for human identification in different continental populations. *Forensic Sci Int Genet.* 32 18–25. 10.1016/j.fsigen.2017.09.012 29024923

[B83] Pereira R. Gomes I. Amorim A. Gusmão L. (2007). Genetic diversity of 10 X chromosome STRs in northern Portugal. *Int. J. Legal Med.* 121 192–197. 10.1007/s00414-006-0144-4 17206433

[B84] Pereira R. Pereira V. Gomes I. Tomas C. Morling N. Amorim A. (2012). A method for the analysis of 32 X chromosome insertion deletion polymorphisms in a single PCR. *Int J Legal Med.* 126 97–105. 10.1007/s00414-011-0593-2 21717151

[B85] Pereira R. Phillips C. Alves C. Amorim A. Carracedo A. Gusmão L. (2009). A new multiplex for human identification using insertion/deletion polymorphisms. *Electrophoresis.* 30 3682–3690. 10.1002/elps.200900274 19862748

[B86] Pereira V. Gusmão L. (2016). “Types of Genomes, Sequences and Genetic Markers (Repeats, SNPs, Indels, Haplotypes),” in *Handbook Of Forensic Genetics: Biodiversity And Heredity In Civil And Criminal Investigation*, Vol. 2 (Singapore: World Scientific), 163 10.1142/9781786340788_0009

[B87] Petkovski E. Keyser-Tracqui C. Hienne R. Ludes B. (2005). SNPs and MALDI-TOF MS: tools for DNA typing in forensic paternity testing and anthropology. *J Forensic Sci.* 50 535–541. 15932083

[B88] Phillips C. Ballard D. Gill P. Syndercombe Court D. Carracedo A. Lareu M. V. (2012). The recombination landscape around forensic STRs: Accurate measurement of genetic distances between syntenic STR pairs using HapMap high density SNP data. *Forensic Science International: Genetics* 6 354–365. 10.1016/j.fsigen.2011.07.012 21871851

[B89] Phillips C. Devesse L. Ballard D. van Weert L. de la Puente M. Melis S. (2018). Global patterns of STR sequence variation: Sequencing the CEPH human genome diversity panel for 58 forensic STRs using the Illumina ForenSeq DNA Signature Prep Kit. *Electrophoresis* 39 2708–2724. 10.1002/elps.201800117 30101987

[B90] Pinto N. Gusmão L. Amorim A. (2011a). X-chromosome markers in kinship testing: a generalisation of the IBD approach identifying situations where their contribution is crucial. *Forensic Sci. Int. Genet.* 5 27–32. 10.1016/j.fsigen.2010.01.011 20457080

[B91] Pinto N. Gusmão L. Silva P. V. Amorim A. (2011b). Estimating coancestry from genotypes using a linear regression method. *Forensic Science International: Genetics Supplement Series* 3 e373–e374. 10.1016/j.fsigss.2011.09.048

[B92] Pinto N. Gusmão L. Egeland L. T. Amorim A. (2013a). Paternity exclusion power: comparative behaviour of autosomal and X-chromosomal markers in standard and deficient cases with inbreeding. *Forensic Sci. Int. Genet.* 7 290–295. 10.1016/j.fsigen.2012.12.002 23312390

[B93] Pinto N. Gusmão L. Egeland T. Amorim A. (2013b). Estimating relatedness with no prior specification of any genealogy: The role of the X-chromosome. *Forensic Science International: Genetics Supplement Series* 4 e252–e253. 10.1016/j.fsigss.2013.10.129

[B94] Pinto N. Silva P. V. Amorim A. (2010). General Derivation of the Sets of Pedigrees with the Same Kinship Coefficients. *Hum. Hered.* 70 194–204. 10.1159/000316390 20720433

[B95] Pinto N. Silva P. V. Amorim A. (2012). A general method to assess the utility of the X-chromosomal markers in kinship testing. *Forensic Science International: Genetics* 6 198–207. 10.1016/j.fsigen.2011.04.014 21592877

[B96] Prieto-Fernández E. Baeta M. Núñez C. Zarrabeitia M. T. Herrera R. J. (2016). Development of a new highly efficient 17 X-STR multiplex for forensic purposes. *Electrophoresis.* 37 1651–1658. 10.1002/elps.201500546 27060859

[B97] Primmer C. R. Saino N. Møller A. P. Ellegren H. (1996). Directional evolution in germline microsatellite mutations. *Nat. Genet.* 13 391. 10.1038/ng0896-391 8696329

[B98] Ross M. T. Grafham D. V. Coffey A. J. Scherer S. McLay K. Muzny D. (2005). The DNA sequence of the human X chromosome. *Nature* 434 325–337. 10.1038/nature03440 15772651 PMC2665286

[B99] Schaffner S. F. (2004). The X chromosome in population genetics. *Nat. Rev. Genet.* 5 43–51. 10.1038/nrg1247 14708015

[B100] Schlötterer C. (2000). Evolutionary dynamics of microsatellite DNA. *Chromosoma* 109 365–371. 10.1007/s004120000089 11072791

[B101] Schlötterer C. Tautz D. (1992). Slippage synthesis of simple sequence DNA. *Nucleic Acids Res* 20 211–215. 10.1093/nar/20.2.211 1741246 PMC310356

[B102] Shimmin L. C. Chang B. H. Li W. H. (1993). Male-driven evolution of DNA sequences. *Nature* 362 745–747. 10.1038/362745a0 8469284

[B103] Sleddens H. F. Oostra B. A. Brinkmann A. O. Trapman J. (1992). Trinucleotide repeat polymorphism in the androgen receptor gene (AR). *Nucleic Acids Res.* 20 1427. 10.1093/nar/20.6.1427-a 1561105 PMC312201

[B104] Stepanov V. Vagaitseva K. Kharkov V. Cherednichenko A. Bocharova A. Berezina G. (2016). Forensic and population genetic characteristics of 62 X chromosome SNPs revealed by multiplex PCR and MALDI-TOF mass spectrometry genotyping in 4 North Eurasian populations. *Leg Med (Tokyo).* 18 66–71. 10.1016/j.legalmed.2015.12.008 26832380

[B105] Strand M. Prolla T. A. Liskay R. M. Petes T. D. (1993). Destabilization of tracts of simple repetitive DNA in yeast by mutations affecting DNA mismatch repair. *Nature* 365 274. 10.1038/365274a0 8371783

[B106] STRidER. (2020). *(STRs for Identity ENFSI Reference Database).* Avaliable at: https://strider.online/ (accessed May 10, 2020).

[B107] Szibor R. (2007). X-chromosomal markers: past, present and future. *Forensic Sci Int Genet* 1 93–99. 10.1016/j.fsigen.2007.03.003 19083736

[B108] Szibor R. Edelmann J. Hering S. Gomes I. Gusmão L. (2009). Nomenclature Discrepancies in the HPRTB Short Tandem Repeat. *Int. J. Legal Med.* 123 185–186. 10.1007/s00414-008-0314-7 19137322

[B109] Szibor R. Hering S. Edelmann J. (2005). The HumARA genotype is linked to spinal and bulbar muscular dystrophy and some further disease risks and should no longer be used as a DNA marker for forensic purposes. *Int. J. Legal Med.* 119 179–180. 10.1007/s00414-005-0525-0 15690183

[B110] Szibor R. Hering S. Edelmann J. (2006). A new Web site compiling forensic chromosome X research is now online. *Int J Legal Med.* 120 252–254. 10.1007/s00414-005-0029-y 16133565

[B111] Szibor R. Krawczak M. Hering S. Edelmann J. Kuhlisch E. Krause D. (2003). Use of X-linked markers for forensic purposes. *Int. J. Legal Med.* 117 67–74. 10.1007/s00414-002-0352-5 12690502

[B112] Tao R. Zhang J. Sheng X. Zhang J. Yang Z. Chen C. (2019). Development and validation of a multiplex insertion/deletion marker panel, SifaInDel 45plex system. *Forensic Science International: Genetics* 41 128–136. 10.1016/j.fsigen.2019.04.008 31079022

[B113] The International HapMap Consortium. (2007). A second generation human haplotype map of over 3.1 *million SNPs*. *Nature* 449 851–861. 10.1038/nature06258 17943122 PMC2689609

[B114] The International Society for Forensic Genetics [ISFG] (2020). Avaliable at: www.isfg.org (accessed May 10, 2020).

[B115] Tillmar A. O. Egeland T. Lindblom B. Holmlund G. Mostad P. (2011). Using X-chromosomal markers in relationship testing: calculation of likelihood ratios taking both linkage and linkage disequilibrium into account. *Forensic Sci. Int. Genet.* 5 506–511. 10.1016/j.fsigen.2010.11.004 21167800

[B116] Tillmar A. O. Kling D. Butler J. M. Parson W. Prinz M. Schneider P. M. (2017). DNA Commission of the International Society for Forensic Genetics (ISFG): Guidelines on the use of X-STRs in kinship analysis. *Forensic Sci Int Genet.* 29 269–275. 10.1016/j.fsigen.2017.05.005 28544956

[B117] Tomas C. Pereira V. Morling N. (2012). Analysis of 12 X-STRs in Greenlanders, Danes and Somalis using Argus X-12. *Int J Legal Med.* 126 121–128. 10.1007/s00414-011-0609-y 21887535

[B118] Tomas C. Sanchez J. J. Castro J. A. Børsting C. Morling N. (2010). Forensic usefulness of a 25 X-chromosome single-nucleotide polymorphism marker set. *Transfusion.* 50 2258–2265. 10.1111/j.1537-2995.2010.02696.x 20492613

[B119] Watanabe G. Umetsu K. Yuasa I. Suzuki T. (2000). DXS10011: a hypervariable tetranucleotide STR polymorphism on the X chromosome. *Int J Legal Med.* 113 249–250. 10.1007/s004149900096 10929244

[B120] Weir B. Anderson A. Hepler A. (2006). Genetic relatedness analysis: modern data and new challenges. *Nat. Rev. Genet.* 7 771–780. 10.1038/nrg1960 16983373

[B121] Xu X. Peng M. Fang Z. (2000). The direction of microsatellite mutations is dependent upon allele length. *Nat. Genet.* 24 396. 10.1038/74238 10742105

[B122] YHRD. (2020). *Y-STR Haplotype Reference Database* (YHRD). Avaliable at: https://yhrd.org/YHRD (accessed May 10, 2020).

[B123] Zarrabeitia M. T. Mijares V. Riancho J. A. (2007). Forensic efficiency of microsatellites and single nucleotide polymorphisms on the X chromosome. *Int J Legal Med.* 121 433–437. 10.1007/s00414-007-0169-3 17436009

[B124] Zaumsegel D. Rothschild M. A. Schneider P. M. (2013). A 21 marker insertion deletion polymorphism panel to study biogeographic ancestry. *Forensic Sci Int Genet.* 7 305–312. 10.1016/j.fsigen.2012.12.007 23352554

[B125] Zhang S. Bian Y. Chen A. Zheng H. Gao Y. Hou Y. (2017a). Developmental validation of a custom panel including 273 SNPs for forensic application using Ion Torrent PGM. *Forensic Sci Int Genet.* 27 50–57. 10.1016/j.fsigen.2016.12.003 27951431

[B126] Zhang S. Lin Y. Bian Y. Li C. (2017b). Parallel sequencing of 60 X-chromosome genetic markers including STRs, SNPs and InDels. *Forensic Science International: Genetics Supplement Series* 6 e317–e319. 10.1016/j.fsigss.2017.09.127

