# Supplementary material for: Twenty Years Later: A Comprehensive Review of the X Chromosome Use in Forensic Genetics
Source: Front Genet. 2020 Sep 17;11:926. doi: 10.3389/fgene.2020.00926 (PMC7527635; doi:10.3389/fgene.2020.00926)
Supplement: Supplementary file 1 [file Table_1.DOCX]

**Supplementary Table 1.** X chromosome specific STR polymorphisms described in the literature till date. The reference given is not the original report, but the most recent usage of the marker. Literature search was performed using Scopus database (https://www.scopus.com/search/form.uri?display=basic), PubMed Central (PMC), U.S. National Institutes of Health's National Library of Medicine. Proceeding reports were found using FSI:Genetics Journal’s website directly.

| **Locus** | **Reference** | **Aliases/ comments** |
| --- | --- | --- |
| DXS6807 | Lin, L., Li, J., Hu, Y. et al. Genetic characterization of 19 X-STRs in Sierra Leone population from Freetown. Int J Legal Med (2020). | DXS9910; GATA52B03; RH28271; RH63404; RH7712 |
| DXS9895 | Deng, C., Song, F., Li, J., Li, Y., Hou, Y., Luo, H. Multiplex PCR for 19 X-chromosomal STRs in Chinese population. Forensic Science International: Genetics Supplement Series December (2017) (6) e24-e26 | P35270; GATA124B04 |
| DXS10148 | He G, Zou X, Wang M et al. Population genetics, diversity, forensic characteristics of four Chinese populations inferred from X-chromosomal short tandem repeats. Legal Medicine (2020) 43:101677 | **-** |
| DXS10135 | Pinto, N, Pereira, V, Tomas, C et al. Paternal and maternal mutations in X-STRs: A GHEP-ISFG collaborative study. Forensic Science International: Genetics (2020) 46:102258 | **-** |
| DXS8378 | Gomes C, Amorim A, Okolie, VO et al. Genetic insight into Nigerian population groups using an X-chromosome decaplex system. Forensic Science International: Genetics Supplement Series (2019) 7(1)501-503 | GATA119E07 |
| DXS9902 | Gomes C, Amorim A, Okolie, VO et al. Genetic insight into Nigerian population groups using an X-chromosome decaplex system. Forensic Science International: Genetics Supplement Series (2019) 7(1)501-503 | GATA175D03 |
| DXS6795 | Yang Z, Chen C, Zhang J et al. Genetic polymorphisms in 16 X-STR loci analyzed in the She population from Zhejiang Province, China Legal Medicine (2019) 39, 25-28 | ATA28C05 |
| DXS9907 | Hering S, Edelmann J, Augustin C, Szibor R, Immel UD. Chromosome X markers DXS6795, DXS9907 and GATA144D04: Repeat structure and allele distribution in a German population. Forensic Science International: Genetics Supplement Series (2011) 3(1), e321-e322 | GATA186D06 |
| DXS6810 | Yang Z, Chen C, Zhang J et al. Genetic polymorphisms in 16 X-STR loci analyzed in the She population from Zhejiang Province, China Legal Medicine (2019) 39, 25-28 | GATA69C12; RH28272; RH30556; RH63417; RH7726; SHGC-18105 |
| GATA144D04 | Deng C, Song F, Li J et al. Multiplex PCR for 19 X-chromosomal STRs in Chinese population. Forensic Science International: Genetics Supplement Series December (2017) (6) e24-e26 | - |
| DXS10076 | Fukuta, M., Gaballah, M., Takada, K et al. Genetic polymorphism of 27 X-chromosomal short tandem repeats in an Egyptian population Legal Medicine (2019) 37:64-66 | **-** |
| DXS10077 | Fukuta, M., Gaballah, M., Takada, K et al. Genetic polymorphism of 27 X-chromosomal short tandem repeats in an Egyptian population Legal Medicine (2019) 37:64-66 | **-** |
| DXS10078 | Fukuta, M., Gaballah, M., Takada, K et al. Genetic polymorphism of 27 X-chromosomal short tandem repeats in an Egyptian population Legal Medicine (2019) 37:64-66 | **-** |
| DXS10161 | Deng C, Song F, Li J et al. Multiplex PCR for 19 X-chromosomal STRs in Chinese population. Forensic Science International: Genetics Supplement Series December (2017) (6) e24-e26 | **-** |
| DXS10160 | Deng C, Song F, Li J et al. Multiplex PCR for 19 X-chromosomal STRs in Chinese population. Forensic Science International: Genetics Supplement Series December (2017) (6) e24-e26 | **-** |
| DXS10159 | Yang Z, Chen C, Zhang J et al. Genetic polymorphisms in 16 X-STR loci analyzed in the She population from Zhejiang Province, China Legal Medicine (2019) 39, 25-28 | **-** |
| DXS10162 | Lin L, Li J, Hu Y et al. Genetic characterization of 19 X-STRs in Sierra Leone population from Freetown. Int J Legal Med (2020). doi.org/10.1007/s00414-019-02243-6 | **-** |
| DXS10163 | Fukuta, M., Gaballah, M., Takada, K et al. Genetic polymorphism of 27 X-chromosomal short tandem repeats in an Egyptian population Legal Medicine (2019) 37:64-66 | **-** |
| DXS10164 | Fukuta, M., Gaballah, M., Takada, K et al. Genetic polymorphism of 27 X-chromosomal short tandem repeats in an Egyptian population Legal Medicine (2019) 37:64-66 | **-** |
| DXS10165 | Elakkary S, Hoffmeister-Ullerich S, Schulze C. Genetic polymorphisms of twelve X-STRs of the investigator Argus X-12 kit and additional six X-STR centromere region loci in an Egyptian population sampleForensic Science International: Genetics (2014) 11(1) 26-30 | **-** |
| DXS7132 | Gomes C, Amorim A, Okolie, VO et al. Genetic insight into Nigerian population groups using an X-chromosome decaplex system. Forensic Science International: Genetics Supplement Series (2019) 7(1)501-503 | GATA72E05; RH28273; RH30545; RH63248; RH7563; SHGC-18086; sWXD2228 |
| DXS10079 | Jiménez- Moreno S, García C, Lobato E, Baeta M, Bañón E. M.de Pancorbo M. Genetic analysis of 12 X-chromosomal STRs an autochthonous population of Southeast Spain. Forensic Science International: Genetics Supplement Series (2019) 7(1) 482-484 | **-** |
| DXS10074 | Jiménez- Moreno S, García C, Lobato E, Baeta M, Bañón E. M.de Pancorbo M. Genetic analysis of 12 X-chromosomal STRs an autochthonous population of Southeast Spain. Forensic Science International: Genetics Supplement Series (2019) 7(1) 482-484 | **-** |
| DXS10075 | Ferragut, J.F., Bassitta, M., Torrens, V et al. Analysis of 21 X-chromosome polymorphisms in urban and rural populations in Salta province (north-western Argentina) International Journal of Legal Medicine (2019) 133(4) 1043-1047 | **-** |
| DXS981 | Fukuta, M., Gaballah, M., Takada, K et al. Genetic polymorphism of 27 X-chromosomal short tandem repeats in an Egyptian population Legal Medicine (2019) 37:64-66 | sWXD528 |
| DXS6800 | Yang Z, Chen C, Zhang J et al. Genetic polymorphisms in 16 X-STR loci analyzed in the She population from Zhejiang Province, China Legal Medicine (2019) 39, 25-28 | GATA31D10; RH28269; RH63257; RH7569 |
| DXS6803 | Yang Z, Chen C, Zhang J et al. Genetic polymorphisms in 16 X-STR loci analyzed in the She population from Zhejiang Province, China Legal Medicine (2019) 39, 25-28 | GATA45H11; RH63401; RH7706 |
| DXS9898 | Zambrano AK, Vaca-Pólit M, Boada, L et al. A X-STR decaplex study in the population of Imbabura-Ecuador. Forensic Science International: Genetics Supplement Series (2019) 7(1)288-290 | GATA126G01 |
| DXS6801 | He G, Zou X, Wang M et al. Population genetics, diversity, forensic characteristics of four Chinese populations inferred from X-chromosomal short tandem repeats. Legal Medicine (2020) 43:101677 | GATA41B11 |
| DXS6809 | He G, Zou X, Wang M et al. Population genetics, diversity, forensic characteristics of four Chinese populations inferred from X-chromosomal short tandem repeats. Legal Medicine (2020) 43:101677 | GATA69B12; RH30555; RH63263; RH7574; SHGC-18104 |
| DXS6789 | He G, Zou X, Wang M et al. Population genetics, diversity, forensic characteristics of four Chinese populations inferred from X-chromosomal short tandem repeats. Legal Medicine (2020) 43:101677 | CHLC.32036; GATA31F01; RH28266; RH34061; SHGC-4389 |
| DXS6799 | Fukuta, M., Gaballah, M., Takada, K et al. Genetic polymorphism of 27 X-chromosomal short tandem repeats in an Egyptian population Legal Medicine (2019) 37:64-66 | GATA29G07; RH28268; RH30550; RH63603; RH7691; SHGC-18097 |
| DXS7424 | He G, Zou X, Wang M et al. Population genetics, diversity, forensic characteristics of four Chinese populations inferred from X-chromosomal short tandem repeats. Legal Medicine (2020) 43:101677 | **-** |
| DXS101 | Fukuta, M., Gaballah, M., Takada, K et al. Genetic polymorphism of 27 X-chromosomal short tandem repeats in an Egyptian population Legal Medicine (2019) 37:64-66 | sWXD1232; |
| DXS6797 | Deng C, Song F, Li J et al. Multiplex PCR for 19 X-chromosomal STRs in Chinese population. Forensic Science International: Genetics Supplement Series December (2017) (6) e24-e26 | GATA10C11; RH28267; RH63601; RH7688 |
| DXS7133 | Zambrano AK, Vaca-Pólit M, Boada, L et al. A X-STR decaplex study in the population of Imbabura-Ecuador. Forensic Science International: Genetics Supplement Series (2019) 7(1)288-290 | GATA81B07; RH30546; RH63329; RH7636; RH95783; SHGC-18089 |
| DXS6804 | Deng C, Song F, Li J et al. Multiplex PCR for 19 X-chromosomal STRs in Chinese population. Forensic Science International: Genetics Supplement Series December (2017) (6) e24-e26 | GATA46G10; RH28270; RH30553; RH63403; RH7709; SHGC-18101 |
| GATA172D05 | Yang Z, Chen C, Zhang J et al. Genetic polymorphisms in 16 X-STR loci analyzed in the She population from Zhejiang Province, China Legal Medicine (2019) 39, 25-28 | **-** |
| DXS7130 | Fukuta, M., Gaballah, M., Takada, K et al. Genetic polymorphism of 27 X-chromosomal short tandem repeats in an Egyptian population Legal Medicine (2019) 37:64-66 | GATA119G08; sWXD2629 |
| GATA165B12 | Yang Z, Chen C, Zhang J et al. Genetic polymorphisms in 16 X-STR loci analyzed in the She population from Zhejiang Province, China Legal Medicine (2019) 39: 25-28 | **-** |
| DXS10103 | Jiménez- Moreno S, García C, Lobato E, Baeta M, Bañón E. M.de Pancorbo M. Genetic analysis of 12 X-chromosomal STRs an autochthonous population of Southeast Spain. Forensic Science International: Genetics Supplement Series (2019) 7(1) 482-484 | **-** |
| HPRTB | Chen M, Ren H, Liu Z et al. Genetic polymorphisms and mutation rates of 16 X-STRs in a Han Chinese population of Beijing and application examples in second-degree kinship cases. International Journal of Legal Medicine (2020) 134(1)163-168 | **-** |
| DXS10101 | Jiménez- Moreno S, García C, Lobato E, Baeta M, Bañón E. M.de Pancorbo M. Genetic analysis of 12 X-chromosomal STRs an autochthonous population of Southeast Spain. Forensic Science International: Genetics Supplement Series (2019) 7(1) 482-484 | **-** |
| GATA31E08 | Zambrano AK, Vaca-Pólit M, Boada, L et al. A X-STR decaplex study in the population of Imbabura-Ecuador. Forensic Science International: Genetics Supplement Series (2019) 7(1) 288-290 | CHLC.31776; GATA-D3S2390; RH28260 |
| DXS9908 | Edelmann J, Lessig R, Willenberg A et al. Forensic validation of the X-chromosomal STR-markers GATA165B12, GATA164A09, DXS9908 and DXS7127 in German population. International Congress Series (2006) 1288: 298-300 | GATA182E04 |
| DXS7127 | Edelmann J, Lessig R, Willenberg A et al. Forensic validation of the X-chromosomal STR-markers GATA165B12, GATA164A09, DXS9908 and DXS7127 in German population. International Congress Series (2006) 1288: 298-300 | GATA100G03;DXS9894; GATA-P33734; RH30548; RH63677; RH7770; SHGC-18092 |
| DXS8377 | Fukuta, M., Gaballah, M., Takada, K et al. Genetic polymorphism of 27 X-chromosomal short tandem repeats in an Egyptian population Legal Medicine (2019) 37:64-66 | **-** |
| DXS10146 | Jiménez- Moreno S, García C, Lobato E, Baeta M, Bañón E. M.de Pancorbo M. Genetic analysis of 12 X-chromosomal STRs an autochthonous population of Southeast Spain. Forensic Science International: Genetics Supplement Series (2019) 7(1) 482-484 | **-** |
| DXS10134 | Jiménez- Moreno S, García C, Lobato E, Baeta M, Bañón E. M.de Pancorbo M. Genetic analysis of 12 X-chromosomal STRs an autochthonous population of Southeast Spain. Forensic Science International: Genetics Supplement Series (2019) 7(1) 482-484 | **-** |
| DXS10147 | Ferragut, J.F., Bassitta, M., Torrens, V et al. Analysis of 21 X-chromosome polymorphisms in urban and rural populations in Salta province (north-western Argentina) International Journal of Legal Medicine (2019) 133(4) 1043-1047 | **-** |
| DXS7423 | Zambrano AK, Vaca-Pólit M, Boada, L et al. A X-STR decaplex study in the population of Imbabura-Ecuador. Forensic Science International: Genetics Supplement Series (2019) 7(1) 288-290 | sWXD2772 |
| DXS6854 | Liu Q-L, Li Z-D, Li C-T et al. X chromosomal recombination - A family study analyzing 26 X-STR Loci in Chinese Han three-generation pedigrees Electrophoresis (2013) 34(20-21) 3016-3022 | **-** |
| DXS6808 | X-chromosome genetic variation in São Paulo State (Brazil) population Martins, J.A., Silva, R.H.A., Freschi, A., (...), Oliveira, R.N., Cicarelli, R.M.B. 2010 Annals of Human Biology 37(4), pp. 598-603 | GATA68H10 |
| DXS6793 | Israr M, Shahid AA, Rahman Z et al. Development and characterization of a new 12-plex ChrX miniSTR system. International Journal of Legal Medicine 128 (2014) (4) 595-598 | ATA14A02; |
| LC149476 | Nishi T, Fukui K, Iwadate K. Genetic polymorphism analyses of three novel X chromosomal short tandem repeat loci in the Xp22.3 region.  Legal Medicine (2020) 45:101709 | - |
| LC149480 | Nishi T, Fukui K, Iwadate K. Genetic polymorphism analyses of three novel X chromosomal short tandem repeat loci in the Xp22.3 region.  Legal Medicine (2020) 45:101709 | - |
| LC149479 | Nishi T, Fukui K, Iwadate K. Genetic polymorphism analyses of three novel X chromosomal short tandem repeat loci in the Xp22.3 region.  Legal Medicine (2020) 45:101709 | - |
| LC149484 | Nishi T, Fukui K, Iwadate K. Genetic polymorphism analyses of three novel X chromosomal short tandem repeat loci in the Xp22.3 region.  Legal Medicine (2020) 45:101709 | - |
| LC317283 | Nishi T, Fukui K, Iwadate K. Genetic polymorphism analyses of three novel X chromosomal short tandem repeat loci in the Xp22.3 region.  Legal Medicine (2020) 45:101709 | - |
| LC317284 | Nishi T, Fukui K, Iwadate K. Genetic polymorphism analyses of three novel X chromosomal short tandem repeat loci in the Xp22.3 region.  Legal Medicine (2020) 45:101709 | - |
| LC317285 | Nishi T, Fukui K, Iwadate K. Genetic polymorphism analyses of three novel X chromosomal short tandem repeat loci in the Xp22.3 region.  Legal Medicine (2020) 45:101709 | - |
| DXS7129 | Dong C, Fu L, Zhang X et al. Development of three X-linked tetrameric microsatellite markers for forensic purposes. Molecular Biology Reports (2014) 41(10), pp. 6429-6432 | GATA117G02; GATA-P34273; sWXD2877 |
| DXS2500 | Dong C, Fu L, Zhang X et al. Development of three X-linked tetrameric microsatellite markers for forensic purposes. Molecular Biology Reports (2014) 41(10), pp. 6429-6432 | HUMUT1595; sWXD1688 |
| G10583 | Dong C, Fu L, Zhang X et al. Development of three X-linked tetrameric microsatellite markers for forensic purposes. Molecular Biology Reports (2014) 41(10), pp. 6429-6432 | GATA151A05 |
| DXS10102 | Samejima M, Nakamura Y, Minaguchi K. Population genetic study of six closely linked groups of X-STRs in a Japanese population. Int J Legal Med. 2011 Nov;125(6):895-900. | - |
| DXS10106 | Samejima M, Nakamura Y, Minaguchi K. Population genetic study of six closely linked groups of X-STRs in a Japanese population. Int J Legal Med. 2011 Nov;125(6):895-900. | - |
| DXS680705 | Lee J C-I, Lin C-Y, Tsai L-C et al. Establishment of 11 linked X-STR loci within 1.1 Mb to assist with kinship testing. International Journal of Legal Medicine (2017) 132(4): 967–973. | - |
| DXS680707 | Lee J C-I, Lin C-Y, Tsai L-C et al. Establishment of 11 linked X-STR loci within 1.1 Mb to assist with kinship testing. International Journal of Legal Medicine (2017) 132(4): 967–973. | - |
| DXS680708 | Lee J C-I, Lin C-Y, Tsai L-C et al. Establishment of 11 linked X-STR loci within 1.1 Mb to assist with kinship testing. International Journal of Legal Medicine (2017) 132(4): 967–973. | - |
| DXS680709 | Lee J C-I, Lin C-Y, Tsai L-C et al. Establishment of 11 linked X-STR loci within 1.1 Mb to assist with kinship testing. International Journal of Legal Medicine (2017) 132(4): 967–973. | - |
| DXS680704 | Lee J C-I, Lin C-Y, Tsai L-C et al. Establishment of 11 linked X-STR loci within 1.1 Mb to assist with kinship testing. International Journal of Legal Medicine (2017) 132(4): 967–973. | - |
| DXS680710 | Lee J C-I, Lin C-Y, Tsai L-C et al. Establishment of 11 linked X-STR loci within 1.1 Mb to assist with kinship testing. International Journal of Legal Medicine (2017) 132(4): 967–973. | - |
| DXS680701 | Lee J C-I, Lin C-Y, Tsai L-C et al. Establishment of 11 linked X-STR loci within 1.1 Mb to assist with kinship testing. International Journal of Legal Medicine (2017) 132(4): 967–973. | - |
| DXS680703 | Lee J C-I, Lin C-Y, Tsai L-C et al. Establishment of 11 linked X-STR loci within 1.1 Mb to assist with kinship testing. International Journal of Legal Medicine (2017) 132(4): 967–973. | - |
| DXS680706 | Lee J C-I, Lin C-Y, Tsai L-C et al. Establishment of 11 linked X-STR loci within 1.1 Mb to assist with kinship testing. International Journal of Legal Medicine (2017) 132(4): 967–973. | - |
| DXS680702 | Lee J C-I, Lin C-Y, Tsai L-C et al. Establishment of 11 linked X-STR loci within 1.1 Mb to assist with kinship testing. International Journal of Legal Medicine (2017) 132(4): 967–973. | - |
| DXS-p11.3 | Zhu YS, Wu H, Lai JH. Structure and polymorphism of novel X-chromosome short tandem repeat loci in a Chinese Han population. Genet Mol Res. 2015;14(4):15044‐15049 | - |
| DXS-q12 | Zhu YS, Wu H, Lai JH. Structure and polymorphism of novel X-chromosome short tandem repeat loci in a Chinese Han population. Genet Mol Res. 2015;14(4):15044‐15049 | - |
| DXS-q13.3 | Zhu YS, Wu H, Lai JH. Structure and polymorphism of novel X-chromosome short tandem repeat loci in a Chinese Han population. Genet Mol Res. 2015;14(4):15044‐15049 | - |
| DXS-q22.1 | Zhu YS, Wu H, Lai JH. Structure and polymorphism of novel X-chromosome short tandem repeat loci in a Chinese Han population. Genet Mol Res. 2015;14(4):15044‐15049 | - |
| DXS-q25 | Zhu YS, Wu H, Lai JH. Structure and polymorphism of novel X-chromosome short tandem repeat loci in a Chinese Han population. Genet Mol Res. 2015;14(4):15044‐15049 | - |
| DXS10011 | Yang X, Zhang X, Zhu J et al. Genetic analysis of 19 X chromosome STR loci for forensic purposes in four Chinese ethnic groups. Sci Rep. (2017) 7:42782. | DYS384; HUMUT413 |
| DXS337 | Valle Y, Padilla-Gutiérrez JR, Rodarte K et al. Five X-chromosome short tandem repeats in a Western Mexican population. Clin Chem Lab Med. (2008) 46(10):1388-1390. | sWXD1239 |
| DXS1214 | Yu B, Zhang H, Li S. X-chromosome STRs polymorphisms of Han ethnic group from Northwest China. Forensic Sci Int. (2005) 153(2-3):269-271. | - |
| GATA164A09 | Edelmann J, Lessig R, Willenberg A et al. Forensic validation of the X-chromosomal STR-markers GATA165B12, GATA164A09, DXS9908 and DXS7127 in German population. International Congress Series (2006) 1288 298-300 | - |
