# Supplementary material for: Twenty Years Later: A Comprehensive Review of the X Chromosome Use in Forensic Genetics
Source: Front Genet. 2020 Sep 17;11:926. doi: 10.3389/fgene.2020.00926 (PMC7527635; doi:10.3389/fgene.2020.00926)
Supplement: Supplementary file 3 [file Table_3.DOCX]

Supplementary Table 3. Compilation of marker mutation rates per familial configuration and respective confidence interval (95%) reported in the literature.

|  | Mother-son duos | | | | Mother-daughter duos | | | | Father-daughter duos | | | | Trios with daughter | | | |  |
| --- | --- | --- | --- | --- | --- | --- | --- | --- | --- | --- | --- | --- | --- | --- | --- | --- | --- |
| Markers | Meioses | Mutations | Mutation Rate | CI (95%) | Meioses | Mutations | Mutation Rate | CI (95%) | Meioses | Mutations | Mutation Rate | CI (95%) | Meioses | Mutations | Mutation Rate | CI (95%) | References |
| DXS101 | 737 | 0 | 0,000 | 0,000-0,005 | 40 | 0 | 0,000 | 0,000-0,088 | 33 | 0 | 0,000 | 0,000-0,106 | 1021 | 1 | 0,001 | 0,000-0,005 | [1, 4, 7, 9, 12, 16, 18] |
| DXS7424 | 940 | 0 | 0,000 | 0,000-0,004 | 335 | 0 | 0,000 | 0,000-0,011 | 186 | 0 | 0,000 | 0,000-0,020 | 975 | 2 | 0,002 | 0,000-0,007 | [2, 7, 12, 16, 18, 20] |
| DXS6803 | 1034 | 0 | 0,000 | 0,000-0,004 | 335 | 0 | 0,000 | 0,000-0,011 | 165 | 0 | 0,000 | 0,000-0,022 | 847 | 3 | 0,004 | 0,001-0,010 | [3, 10, 18, 20] |
| DXS9895 | - | - | - | - | - | - | - | - | - | - | - | - | 78 | 0 | 0,000 | 0,000-0,046 | [3] |
| DXS8377 | 77 | 1 | 0,013 | 0,000-0,070 | - | - | - | - | 12 | 0 | 0,000 | 0,000-0,265 | 134 | 1 | 0,007 | 0,000-0,041 | [4, 8, 9, 12] |
| HPRTB | 904 | 1 | 0,001 | 0,000-0,006 | 335 | 1 | 0,003 | 0,000-0,017 | 329 | 3 | 0,009 | 0,002-0,026 | 2946 | 7 | 0,002 | 0,001-0,005 | [4, 6, 7, 8, 9, 11, 12, 14, 15, 18, 20, 21] |
| DXS7132 | 1332 | 1 | 0,001 | 0,000-0,004 | 335 | 1 | 0,003 | 0,000-0,017 | 329 | 2 | 0,006 | 0,001-0,022 | 3577 | 32 | 0,009 | 0,006-0,013 | [5, 6, 7, 8, 9, 10, 11, 12, 14, 15, 16, 17, 18, 19, 20, 21] |
| DXS7133 | 177 | 0 | 0,000 | 0,000-0,021 | - | - | - | - | 21 | 0 | 0,000 | 0,000-0,161 | 474 | 0 | 0,000 | 0,000-0,008 | [5, 7, 12, 13, 16] |
| GATA172D05 | 820 | 0 | 0,000 | 0,000-0,004 | 335 | 0 | 0,000 | 0,000-0,011 | 176 | 0 | 0,000 | 0,000-0,021 | 676 | 2 | 0,003 | 0,000-0,011 | [5, 7, 8, 9, 17, 18, 20] |
| DXS8378 | 891 | 2 | 0,002 | 0,000-0,008 | 335 | 0 | 0,000 | 0,000-0,011 | 329 | 0 | 0,000 | 0,000-0,011 | 2933 | 6 | 0,002 | 0,001-0,004 | [6, 7, 8, 9, 11, 12, 14, 15, 17, 18, 19, 20, 21] |
| DXS7423 | 618 | 0 | 0,000 | 0,000-0,006 | 40 | 0 | 0,000 | 0,000-0,088 | 175 | 0 | 0,000 | 0,000-0,021 | 2875 | 4 | 0,001 | 0,000-0,004 | [5, 6, 7, 9, 11, 12, 14, 15, 17, 18, 19, 21] |
| DXS6809 | 498 | 2 | 0,004 | 0,000-0,014 | - | - | - | - | 11 | 0 | 0,000 | 0,000-0,285 | 715 | 4 | 0,006 | 0,002-0,014 | [7, 9, 10, 16, 17] |
| DXS6789 | 1261 | 0 | 0,000 | 0,000-0,003 | 335 | 0 | 0,000 | 0,000-0,011 | 176 | 0 | 0,000 | 0,000-0,021 | 1240 | 5 | 0,004 | 0,001-0,009 | [7, 8, 9, 10, 16, 17, 18, 19, 20] |
| GATA31E08 | 947 | 0 | 0,000 | 0,000-0,004 | 335 | 0 | 0,000 | 0,000-0,011 | 175 | 0 | 0,000 | 0,000-0,021 | 895 | 1 | 0,001 | 0,000-0,006 | [7, 8, 16, 17, 18, 20] |
| DXS6807 | 280 | 0 | 0,000 | 0,000-0,013 | 295 | 0 | 0,000 | 0,000-0,012 | 175 | 0 | 0,000 | 0,000-0,021 | 98 | 0 | 0,000 | 0,000-0,037 | [7, 12, 20] |
| DXS9902 | 777 | 0 | 0,000 | 0,000-0,005 | 335 | 0 | 0,000 | 0,000-0,011 | 165 | 0 | 0,000 | 0,000-0,022 | 545 | 5 | 0,009 | 0,003-0,021 | [8, 17, 18, 20] |
| DXS6810 | 273 | 0 | 0,000 | 0,000-0,013 | 295 | 0 | 0,000 | 0,000-0,012 | 154 | 0 | 0,000 | 0,000-0,024 | 108 | 1 | 0,009 | 0,000-0,051 | [8, 20] |
| DXS981 | 468 | 0 | 0,000 | 0,000-0,008 | - | - | - | - | - | - | - | - | 771 | 0 | 0,000 | 0,000-0,005 | [4, 8, 10, 13, 16] |
| DXS6793 | - | - | - | - | - | - | - | - | - | - | - | - | 50 | 0 | 0,000 | 0,000-0,071 | [8] |
| DXS6801 | 170 | 0 | 0,000 | 0,000-0,021 | - | - | - | - | - | - | - | - | 360 | 0 | 0,000 | 0,000-0,010 | [8, 16] |
| DXS9898 | 234 | 0 | 0,000 | 0,000-0,016 | - | - | - | - | 12 | 0 | 0,000 | 0,000-0,265 | 381 | 0 | 0,000 | 0,000-0,010 | [9, 12, 16, 17] |
| DXS10135 | 64 | 1 | 0,016 | 0,000-0,084 | - | - | - | - | 46 | 1 | 0,022 | 0,001-0,115 | 2297 | 45 | 0,020 | 0,014-0,026 | [11, 14, 15, 19, 21] |
| DXS10074 | 248 | 0 | 0,000 | 0,000-0,015 | - | - | - | - | 46 | 0 | 0,000 | 0,000-0,077 | 2627 | 23 | 0,009 | 0,006-0,013 | [11, 14, 15, 16, 17, 19, 21] |
| DXS10101 | 64 | 0 | 0,000 | 0,000-0,056 | - | - | - | - | 46 | 0 | 0,000 | 0,000-0,077 | 2297 | 14 | 0,006 | 0,003-0,010 | [11, 14, 15, 19, 21] |
| DXS10134 | 351 | 0 | 0,000 | 0,000-0,010 | 295 | 0 | 0,000 | 0,000-0,012 | 200 | 1 | 0,005 | 0,000-0,028 | 2375 | 21 | 0,009 | 0,005-0,013 | [11, 14, 15, 17, 19, 20, 21] |
| DXS6800 | 450 | 0 | 0,000 | 0,000-0,008 | 295 | 0 | 0,000 | 0,000-0,012 | 165 | 0 | 0,000 | 0,000-0,022 | 452 | 0 | 0,000 | 0,000-0,008 | [12, 13, 16, 20] |
| DXS6797 | - | - | - | - | - | - | - | - | - | - | - | - | 84 | 0 | 0,000 | 0,000-0,043 | [13] |
| GATA165B12 | 933 | 0 | 0,000 | 0,000-0,004 | 335 | 0 | 0,000 | 0,000-0,011 | 165 | 0 | 0,000 | 0,000-0,022 | 869 | 0 | 0,000 | 0,000-0,004 | [13, 16, 18, 20] |
| DXS10148 | 64 | 0 | 0,000 | 0,000-0,056 | - | - | - | - | 46 | 0 | 0,000 | 0,000-0,077 | 2069 | 19 | 0,009 | 0,006-0,014 | [14, 19, 21] |
| DXS10079 | 234 | 1 | 0,004 | 0,000-0,024 | - | - | - | - | 46 | 0 | 0,000 | 0,000-0,077 | 2379 | 23 | 0,010 | 0,006-0,014 | [14, 16, 19, 21] |
| DXS10103 | 64 | 0 | 0,000 | 0,000-0,056 | - | - | - | - | 46 | 0 | 0,000 | 0,000-0,077 | 2069 | 9 | 0,004 | 0,002-0,008 | [14, 19, 21] |
| DXS10146 | 64 | 0 | 0,000 | 0,000-0,056 | - | - | - | - | 46 | 1 | 0,022 | 0,001-0,115 | 2069 | 13 | 0,006 | 0,003-0,011 | [14, 19, 21] |
| DXS10075 | 170 | 0 | 0,000 | 0,000-0,021 | - | - | - | - | - | - | - | - | 310 | 3 | 0,010 | 0,002-0,028 | [16] |
| DXS10147 | 504 | 0 | 0,000 | 0,000-0,007 | 40 | 0 | 0,000 | 0,000-0,088 | 11 | 0 | 0,000 | 0,000-0,285 | 437 | 0 | 0,000 | 0,000-0,008 | [17, 18] |
| DXS6795 | 763 | 0 | 0,000 | 0,000-0,005 | 335 | 0 | 0,000 | 0,000-0,011 | 165 | 0 | 0,000 | 0,000-0,022 | 475 | 0 | 0,000 | 0,000-0,008 | [18, 19] |
| DXS7130 | 490 | 0 | 0,000 | 0,000-0,008 | 40 | 0 | 0,000 | 0,000-0,088 | 11 | 0 | 0,000 | 0,000-0,285 | 417 | 0 | 0,000 | 0,000-0,009 | [18] |
| DXS10159 | 273 | 0 | 0,000 | 0,000-0,013 | 295 | 0 | 0,000 | 0,000-0,012 | 154 | 1 | 0,00649 | 0,000-0,036 | 58 | 0 | 0,000 | 0,000-0,062 | [20] |

NOTE: Mutations rates were calculated based on the following references: [1] Edelmann *et al.*, 2001; [2] Edelmann *et al.*, 2002; [3] Huang *et al.*, 2003; [4] Athanasiadou *et al.*, 2003; [5] Turrina *et al.*, 2004; [6] Zalan *et al.*, 2007; [7] Turrina *et al.*, 2007; [8] Tariq *et al.*, 2008; [9] Pico *et al.*, 2008; [10] Liu *et al.*, 2008; [11] Becker *et al.*, 2008; [12] Poetsch *et al.*, 2009; [13] Nadeem *et al.*, 2009; [14] Pamjav *et al.*, 2011; [15] Teztaff *et al.*, 2012; [16] Liu *et al.*, 2012; [17] Nishi *et al.*, 2013; [18] Diegoli *et al.*, 2014; [19] García *et al.*, 2018; [20] Chen *et al.*, 2019; [21] Pinto *et al.*, 2020

Athanasiadou, D., *et al.*, (2003). Development of a quadruplex PCR system for the genetic analysis of X-chromosomal STR loci. In *International Congress Series* (Vol. 1239, pp. 311-314). Elsevier.

Becker, D., *et al.*, (2008). Population genetic evaluation of eight X-chromosomal short tandem repeat loci using Mentype Argus X-8 PCR amplification kit. *Forensic Science International: Genetics*, *2*(1), 69-74.

Chen, M., *et al.*, (2020). Genetic polymorphisms and mutation rates of 16 X-STRs in a Han Chinese population of Beijing and application examples in second-degree kinship cases. *International journal of legal medicine*, *134*(1), 163-168.

Diegoli, T. M., *et al.*, (2014). Mutation rates of 15 X chromosomal short tandem repeat markers. *International journal of legal medicine*, *128*(4), 579-587.

Edelmann, J., & Szibor, R. (2001). DXS101: a highly polymorphic X-linked STR. *International journal of legal medicine*, *114*(4-5), 301-304.

Edelmann, J., *et al.*, (2002). Validation of the STR DXS7424 and the linkage situation on the X-chromosome. *Forensic science international*, *125*(2-3), 217-222.

García, M. G., *et al.*, (2019). X-chromosome data for 12 STRs: Towards an Argentinian database of forensic haplotype frequencies. *Forensic Science International: Genetics*, *41*, e8-e13.

Huang, D., *et al.*, (2003). Development of the X-linked tetrameric microsatellite markers HumDXS6803 and HumDXS9895 for forensic purpose. *Forensic science international*, *133*(3), 246-249.

Liu, Q. L., *et al.*, (2008). Development of a five ChX STRs loci typing system. *International journal of legal medicine*, *122*(3), 261-265.

Liu, Q. L., *et al.*, (2012). Development of multiplex PCR system with 15 X‐STR loci and genetic analysis in three nationality populations from C hina. *Electrophoresis*, *33*(8), 1299-1305.

Nadeem, A., *et al.*, (2009). Development of pentaplex PCR and genetic analysis of X chromosomal STRs in Punjabi population of Pakistan. *Molecular biology reports*, *36*(7), 1671-1675.

Nishi, T., *et al.*, (2013). Application of a novel multiplex polymerase chain reaction system for 12 X-chromosomal short tandem repeats to a Japanese population study. *Legal Medicine*, *15*(1), 43-46.

Pamjav, H., *et al.*, (2012). X chromosomal recombination study in three-generation families in Hungary. *Forensic Science International: Genetics*, *6*(3), e95-e96.

Pico, A., *et al.*, (2008). Genetic profile characterization and segregation analysis of 10 X-STRs in a sample from Santander, Colombia. *International journal of legal medicine*, *122*(4), 347-351.

Pinto, N., *et al.*, (2020). Paternal and maternal mutations in X-STRs: A GHEP-ISFG collaborative study. *Forensic Science International: Genetics*, *46*, 102258.

Poetsch, M., *et al.*, (2009). Allele frequencies of 11 X-chromosomal loci in a population sample from Ghana. *International journal of legal medicine*, *123*(1), 81.

Tariq, M. A., *et al.*, (2008). Allele frequency distribution of 13 X-chromosomal STR loci in Pakistani population. *International journal of legal medicine*, *122*(6), 525-528.

Tetzlaff, S., Wegener, R., & Lindner, I. (2012). Population genetic investigation of eight X-chromosomal short tandem repeat loci from a northeast German sample. *Forensic Science International: Genetics*, *6*(6), e155-e156.

Turrina, S., & De Leo, D. (2004, April). Population genetic comparisons of three X-chromosomal STRs (DXS7132, DXS7133 and GATA172D05) in North and South Italy. In *International Congress Series* (Vol. 1261, pp. 302-304). Elsevier.

Turrina, S., *et al.*, (2007). Development and forensic validation of a new multiplex PCR assay with 12 X-chromosomal short tandem repeats. *Forensic Science International: Genetics*, *1*(2), 201-204.

Zalán, A., *et al.*, (2007). Hungarian population data of four X-linked markers: DXS8378, DXS7132, HPRTB, and DXS7423. *International journal of legal medicine*, *121*(1), 74-77.
